# Comparative transcriptomics reveals epigenetic and transcriptional regulatory genes associated with drought stress memory in winter wheat

**DOI:** 10.1093/jxb/erag203

**Published:** 2026-04-29

**Authors:** Carolyn Mukiri Kambona, Michael Schneider, Fei He, Jens Léon, Annaliese S Mason, Agim Ballvora

**Affiliations:** Plant Breeding Department, Institute of Crop Science and Resource Conservation (INRES), University of Bonn, D-53115 Bonn, Germany; Research Institute of Organic Agriculture (FiBL), Frick, Aargau, Switzerland; Plant Breeding Department, Institute of Crop Science and Resource Conservation (INRES), University of Bonn, D-53115 Bonn, Germany; Plant Breeding Department, Institute of Crop Science and Resource Conservation (INRES), University of Bonn, D-53115 Bonn, Germany; Field Lab Campus Klein-Altendorf, Rheinische Friedrich-Wilhelms-University, Bonn, Germany; Plant Breeding Department, Institute of Crop Science and Resource Conservation (INRES), University of Bonn, D-53115 Bonn, Germany; Plant Breeding Department, Institute of Crop Science and Resource Conservation (INRES), University of Bonn, D-53115 Bonn, Germany; CIMMYT, Mexico

**Keywords:** Drought memory, epigenetics, histone modification, intergenerational and transgenerational memory, memory genes, transcriptional memory

## Abstract

Wheat is a staple crop vital to global food security, yet its productivity is increasingly threatened by drought. The extent to which contrasting wheat genotypes exhibit stress memory, and how this shapes responses to repeated drought, remains unclear. We investigated intergenerational and transgenerational drought memory in drought-tolerant ‘Intro’ and drought-sensitive ‘Sonalika’ using integrated morpho-physiological and transcriptomic analyses. Under repeated stress, seedlings of both genotypes with drought history showed enhanced resilience. Transcriptome profiling identified drought memory genes exhibiting consistent differential expression between initial and repeated stress exposures in seeds and leaves. Gene Ontology analysis revealed enrichment of ‘Intro’ seed memory genes in transcripts linked to H3K36me3, while leaf memory genes were enriched in pathways related to abscisic acid signaling, osmoprotection, protein synthesis, and antioxidant activity. By contrast, ‘Sonalika’ showed enrichment of seed transcripts associated with H3K9ac and H3K14ac, and leaf memory genes linked to transcriptional activity, proteostasis, and immune pathways. The functional drought memory exhibited by ‘Sonalika’, despite the absence of transcripts associated with canonical methylation-based chromatin marks, suggests alternative regulatory strategies. Together, these results indicate that wheat seedlings exhibit genotype-specific drought stress memory involving distinct transcriptional programs and putative epigenetic regulators. While histone modifications were not directly assessed, enrichment of histone-related genes suggests their potential involvement, warranting future chromatin-level validation.

## Teaser

Contrasting wheat genotypes exhibit heritable drought stress memory, with differential expression of transcripts associated with histone modification in seeds potentially serving as molecular signals that prime stress-responsive genes for activation during subsequent drought.

## Introduction

Wheat is a primary source of calories and protein for millions globally, with its cultivation spanning diverse climates and regions (Erenstein *et al*., 2022; Gooding and Shewry, 2022). Given its extensive use in food products such as bread, pasta, and other wheat-based goods, ensuring consistent wheat yields is critical for global food security (Curtis and Halford, 2014). However, drought stress can severely affect wheat growth and yield by disrupting crucial physiological processes (Sarto *et al*., 2017; Ahmad *et al*., 2018). Reduced water availability during drought can lower chlorophyll concentration, diminishing photosynthetic efficiency and growth (Xiang *et al*., 2013; Sharma *et al*., 2022). Osmotic adjustment, crucial for maintaining cell turgor under water deficit conditions, is often compromised, affecting root and shoot development and ultimately reducing crop yields (Anjum *et al*., 2011; Sharma *et al*., 2022).

On the other hand, plants, including wheat, react to dehydration through various physiological, biochemical, and morphological changes, often involving cost–benefit compromises (Kozlowski and Pallardy, 2002; Chaves *et al*., 2003). For instance, the rate of photosynthesis, directly correlated with carbon assimilation, is essential for plant growth. During photosynthesis, CO_2_ is absorbed through stomata, enters chloroplasts, and is fixed into stable intermediates that are converted into glucose and other carbohydrates, serving as the building blocks for plant biomass (Lawlor and Cornic, 2002). However, drought-induced increases in the plant hormone abscisic acid (ABA) trigger stomatal closure to reduce water loss, which simultaneously decreases CO_2_ uptake and hampers photosynthesis, illustrating the delicate balance plants must maintain between carbon assimilation and water conservation (Schulze, 1986; Flexas *et al*., 2004, 2007).

While much research has been focused on periodic drought, plants experience repeated drought episodes, sometimes multigenerational and with intervening water recovery. Recent studies suggest that pre-exposure to drought can alter responses to subsequent stress events, a phenomenon known as drought stress memory (Xin and Browse, 2000; Fleta-Soriano and Munné-Bosch, 2016; Abid *et al*., 2018). This memory allows plants to ‘remember’ previous stress and adjust their responses more effectively during future droughts (Jacques *et al*., 2021; Pissolato *et al*., 2024). These drought memory responses could be viewed as coordinated changes resulting from an initial stress encounter, leading to a new homeostatic state (Kartashov *et al*., 2023; Zuo *et al*, 2023; Ganie *et al*., 2024).

Drought stress memory could be classified into somatic, intergenerational, and transgenerational memory, each differing in duration and mode of inheritance (Kambona *et al*., 2023a). Somatic stress memory refers to short-term memory imprints that are mitotically inherited within a single generation, allowing early stress exposure during plant development to prime the plant for improved tolerance to similar stress later in its life cycle (Li and Liu, 2016; Lämke and Bäurle, 2017). Intergenerational stress memory arises when parental plants experience drought stress during reproduction, directly exposing their reproductive cells and seeds to the same environmental conditions. This leads to mitotic transmission of stress cues into the progeny via seed or embryo modifications, thereby influencing their stress responses (Heard and Martienssen, 2014; Lämke and Bäurle, 2017). In contrast, true transgenerational stress memory is observed when stress effects persist beyond the directly exposed generation and are inherited meiotically (Klengel *et al*., 2016). These distinct forms of stress memory shape plant adaptation by regulating physiological and molecular responses (Kambona *et al*., 2023a; Kashyap *et al*., 2024).

Despite this progress, it remains unclear whether and how wheat seedlings exhibit memory of drought experienced by preceding generation(s), and how gene expression changes due to recurrent stress could regulate these responses. Studies in Arabidopsis, soybean, and maize have classified memory genes as those exhibiting altered transcriptional responses upon repeated exposure to stress (Ding *et al*., 2012, 2013, 2014; Kim *et al*., 2020). However, whether such transcriptional memory can be translated into integrated morpho-physiological responses in wheat remains an open question. By comprehending how wheat plants respond to drought at physiological, biochemical, and molecular levels, researchers can develop more resilient wheat varieties, enhance drought tolerance, and ensure stable production in drought-prone areas (Ahmad *et al*., 2018; Mohammadi, 2018).

This study aimed to integrate transcriptome data with morpho-physiological measurements to elucidate the mechanisms of drought stress memory in wheat and identify potential targets for enhancing drought tolerance. Specifically, our objectives were: (i) to determine whether drought stress experienced by previous generations of wheat plants induces changes in the physiological, biochemical, and morphological responses of seedling leaf and root systems in subsequent generations; (ii) to identify gene sets that exhibit coordinated or specific expression changes under different drought histories to pinpoint potential genetic factors contributing to drought stress memory; (ii) to identify statistically over-represented Gene Ontology (GO) terms in these gene sets to uncover key biological pathways, processes, molecular functions, or cellular components relevant to drought memory and stress adaptation; and (iv) to correlate transcriptional and physio-morphological responses to identify candidate genes that may play a central role in mediating drought stress memory.

## Materials and methods

### Plant material and experimental design

**Fig. 1.**
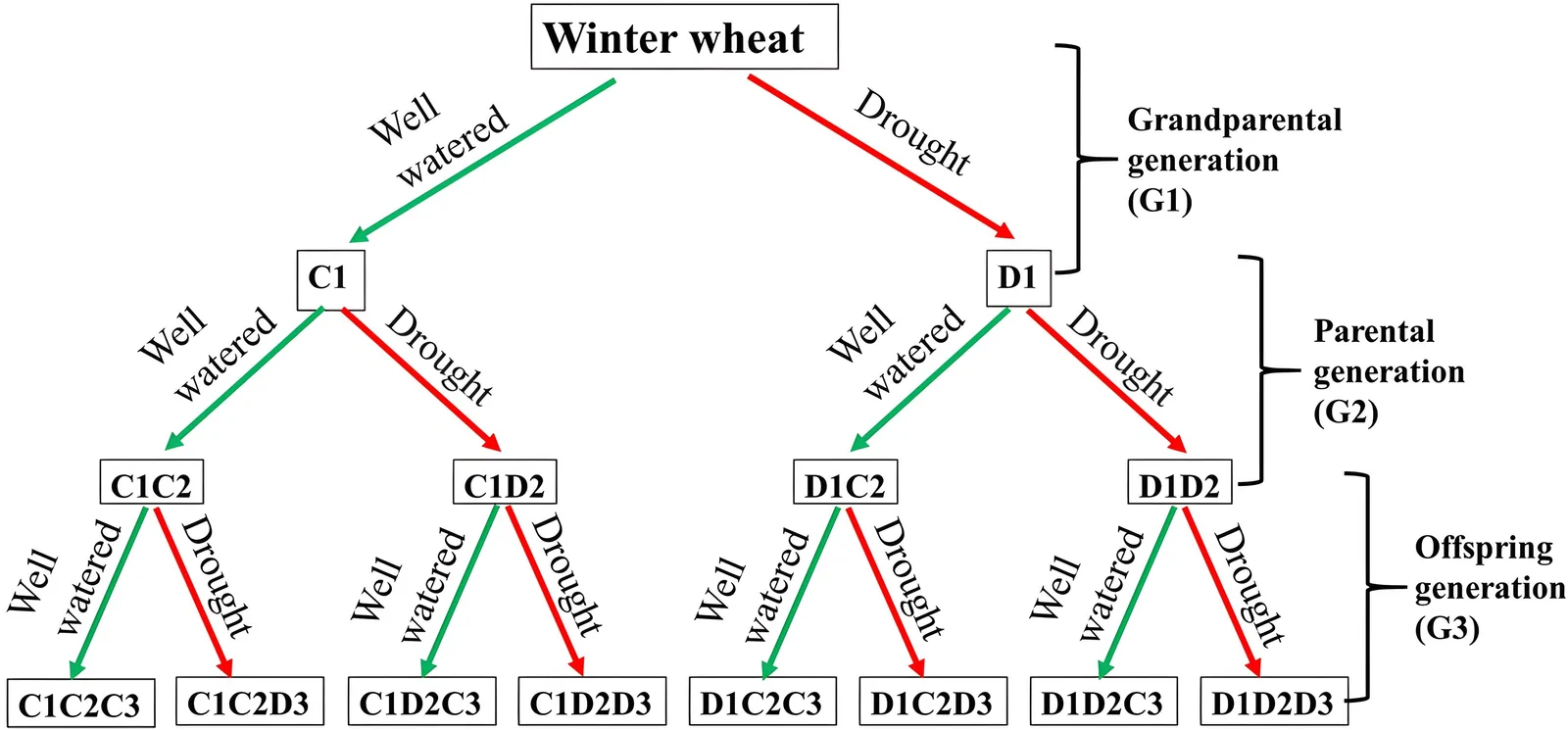
Schematic illustration of the experimental setup showing treatments applied across three consecutive generations (G1, G2, and G3) of winter wheat. In each generation, plants were grown under either well-watered conditions (C) or drought conditions (D). Drought history consists of chronological C or D labels that indicate the treatment applied in each generation, while the numbers 1, 2, and 3 denote the grandparental, parental, and offspring generations, respectively. The design generated plants with no drought history (C1C2C3), one-time drought exposure in different generations (D1C2C3, C1D2C3, C1C2D3), consecutive drought stress over two or three generations (D1D2C3, C1D2D3, D1D2D3), and intermittent drought stress (D1C2D3).

In a previous study, a large population of winter wheat genotypes, representing broad genetic diversity, was screened for the response to drought stress (Koua *et al*., 2021). This population served as the grandparental generation in this study. The selection of two grandparental groups of genotypes, tolerant (six genotypes) and susceptible (nine genotypes) and the cultivation of parental and offspring generations has been published in Kambona *et al*. (2023b). In general, winter wheat genotypes were grown under drought (D) and control (C) treatments for three subsequent generations to produce plants with all possible combinations of drought stress history: (i) only one round of previous drought stress; (ii) drought stress over two or three consecutive generations; and (iii) an intermittent drought stress history (Fig. 1) (Kambona *et al*., 2023b).

### Selection of the two genotypes used in this study

For this study, we randomly selected two genotypes, one each from the tolerant and the susceptible groups. The tolerant genotype, ‘Intro’, was released in 2011, while the drought-sensitive genotype, ‘Sonalika’, was released in 1967. Seed sets collected at the end of the second generation (G2), which included D1D2 (drought stress in G1 and G2), D1C2 (drought stress only in G1), C1D2 (drought stress only in G2), and C1C2 (no history of drought stress treatments), were used (Kambona *et al*., 2023b). A random sample of these seeds were used to generate seed transcriptome data, while the remaining seeds were used to produce seedlings in the following third generation (G3).

### Seedling growth and phenotyping

In the first experimental setup aimed at offspring generation, the four seed sets (D1D2, D1C2, C1D2, and C1C2), were planted for each genotype, each with four repetitions and six seeds per pot. The greenhouse conditions were maintained at 18/14 °C day/night temperatures, with a 16 h photoperiod and a relative humidity of 60–80%. After germination, the seedlings were thinned to four per pot, ensuring uniform spacing. The seedlings were subjected to either drought or control treatments in the fourth week after sowing to have all possible combinations of drought stress history. After 2 weeks of drought application, we measured various physiological and morphological parameters and collected leaf samples for transcriptome analysis under both offspring growth environments (drought and control). The schematic presentation of the experimental design for producing the experimental groups used in this study was previously published in Kambona *et al*. (2023b).

### Measurement of seedling physiological traits

The photochemical quantum yield of PSII was determined using the MINI-PAM-II photosynthesis yield analyzer (Heinz Walz GmbH, Effeltrich, Germany), based on *F* and *F*_M_′ chlorophyll fluorescence on the second leaf of the seedling of each genotype. Gas exchange parameters including stomatal conductance to water (*g*_sw_) and assimilation rate (*A*) were quantified using the LI-6800 fluorometer (LI-6800 Portable Photosynthesis System, Li-Cor Biosciences, Lincoln, NE, USA) on the same leaf of seedlings. Leaf tissue samples were collected and frozen in liquid nitrogen for subsequent analyses of ABA and RNA extraction. ABA concentrations in leaf tissue extracts were quantified using a competitive ELISA kit (Cusabio, Cat. No. CSB-E09159Pl), following the manufacturer’s instructions as described by Asch *et al*. (2001). The kit reports ABA concentrations in µg ml^−1^ of extract, with a sensitivity of <0.04 µg ml^−1^ and a detection range up to 10 µg ml^−1^. To enable direct comparison with ABA concentrations commonly reported on a fresh weight basis, the measured range of <0.04–10 µg ml^−1^ corresponds to ∼9–2200 ng g^−1^ FW based on the measured extraction volume and tissue mass. These converted values are consistent with previously reported endogenous ABA levels in cereals, which typically range from tens to several hundred ng g^−1^ FW under both well-watered and drought stress conditions (Wright, 1969; Quarrie and Henson, 1981; Wang et al., 2022; Zou et al., 2024). For consistency, all ABA values reported herein are presented in the original kit-based units (µg ml^−1^).

### Seedling root morphology

Root architecture parameters were measured in a detailed second experimental setup. The four seed sets (D1D2, D1C2, C1D2, and C1C2) were surface-sterilized using 1% sodium hypochlorite (NaOCl) for 5 min. After washing and drying, 15 seeds per genotype from each seed set were placed on the germination trays, followed by watering and covering with transparent lids. One germinated seed per genotype and seed set was transplanted into tubes filled with 2–3.15 mm Aquagran filter quartz (Euroquarz GmbH, Dorsten, Germany), with three repetitions in two hydroponic systems in the greenhouse. Each hydroponic system was supplied with modified Hoagland nutrient solution as described by Tavakkoli *et al*. (2010), circulated hourly using an EHEIM Universal-pump 1046 (EHEIM GmbH and Co., Deizisau, Germany) and a timer, with weekly nutrient solution changes and pH adjustments to 5.5 initially and then monitored daily and adjusted to 6.0. The greenhouse was illuminated using SOD AGRO 400 W 230 V lightbulbs (DH Licht GmbH, Wülfrath, Germany).

To reduce water potential, after 4 weeks of growth under uniform conditions, one hydroponic system was subjected to a 20% osmotic concentration of polyethylene glycol (PEG-Sagar et al., 2020; Robin et al., 2021). PEG-induced osmotic stress in hydroponics is a widely established approach for drought simulation (Torabi et al., 2012; Osmolovskaya et al., 2018; Sagar et al., 2020; Robin et al., 2021), and is particularly suitable for experiments requiring accurate quantification of root traits, as it ensures uniform exposure across all seedlings and enables recovery of intact root systems without soil-related disturbance. Although PEG does not fully replicate the complexity of field drought such as heterogeneous soil drying and soil–microbe interactions, it provides a reproducible and well-controlled system for comparing genotype-specific responses to defined water-deficit conditions. After 1 week of treatment, seedlings were harvested and preserved in 120 ml plastic labeled containers containing 50% alcohol to completely submerge the roots for longer storage. Root architecture analysis was carried out using an Epson LA2400 pro-optical Scanner (Epson America, Inc., Long Beach, CA, USA) and a WinRHIZO software (Regent Instruments Inc., Quebec City, QC, Canada). The measured traits included seedling root surface area and the number of root crossings.

### Statistical analysis of measured physio-morphological traits under varying drought history

To evaluate the impact of recurrent drought on physiological and morphological traits, we employed a Generalized Linear Mixed-Effects Model (GLMM) ANOVA. Our null hypothesis posited that previous drought stress does not influence the expression of the measured traits under both drought and control environments across the experimental design. The experimental setup included four parental combinations based on prior drought stress history (C1C2, C1D2, D1C2, and D1D2), which were categorized as stress history and treated as a fixed factor. The genotype (‘Intro’ and ‘Sonalika’) was also treated as a fixed factor. Repetition was included as a random factor within the models.

The GLMM was structured as follows:


Yijk=μ+αi+βj+(αβ)ij+v(ab)ij+εijk,

where, Yijk is the response variable (measured trait) in which i indexes the parental group (stress history), j indexes the genotype, and k indexes the observation within each combination of parental group and genotype. μ is the overall mean, αi is the effect of the ith stress history, βj is the effect of the jth genotype, (αβ)ij represents the interaction effect between stress history and genotype, v(ab)ij is the random effect of repetition nested within each combination of stress history and genotype, and εijk is the residual error term (the unexplained variability in the response variable).

Essentially, ANOVA was conducted to assess the significance of the fixed effects (stress history and genotype) on the measured traits, with separate analyses for the control and drought offspring environments. Tukey’s HSD post-hoc tests were applied for pairwise comparisons of the means. The study assumed that seeds from each experimental group were pooled per generation, ensuring that differences in seed pools were solely due to drought treatments in ancestral generations. Significance was set at *P*<0.05 and confidence intervals were set at 95%. All statistical analyses were performed using the ANOVA package in R version 4.3.2 (R Core Team, 2023).

### RNA extraction and Massive Analysis of cDNA Ends sequencing

To examine the effects of ancestral drought on seed and leaf transcriptomes, total RNA was isolated from three independent biological replicates of leaves and seeds corresponding to each drought stress history and genotype. Seed RNA was extracted from the same homogeneous seed lot that was also used for seedling phenotyping, while leaf RNA was obtained from individual seedlings grown from that lot. This strategy ensured that the transcriptomic profiles directly corresponded to the physiological traits measured (Li and Trick, 2005). For the seed samples, the four sets of drought stress history (C1C2, C1D2, D1D2, and D1C2) were analyzed, resulting in a total of 24 Massive Analysis of cDNA Ends (MACE) sequencing samples. In the case of leaf samples, eight sets of drought stress history (C1C2C3, C1C2D3, C1D2C3, C1D2D3, D1C2C3, D1C2D3, D1D2C3, and D1D2D3) were examined, yielding 48 MACE sequencing samples. MACE RNA sequencing was performed by GenXPro GmbH (Frankfurt, Germany). A 3′ mRNA sequencing approach, using Illumina reads of fragments derived from 3′ mRNA ends as described by Zawada *et al*. (2014), was employed to sequence biotinylated 3′-end fragments ranging from 16 bp to 200 bp.

### Bioinformatics analysis

Adapters were removed from the reads using Cutadapt (version 2.10, Martin, 2011). Quality control of the libraries was carried out with FastQC (version 0.11.9), and short reads <35 bp were removed using Trimmomatic (version 0.39, Bolger *et al*., 2014). Across all 72 samples, 221 million single-end short reads were generated, with an average length of 60 bp. Sequencing depth was consistent across biological replicates within each drought history group and genotype, with minimal variation in raw read numbers, filtered reads, or mapped reads, ensuring reliable downstream normalization and differential expression analysis. Sequencing depth was broadly comparable across biological replicates within each drought history group and genotype, with no replicate showing evidence of technical failure. Differences in library size were accounted for during normalization prior to differential expression analysis.

Each library was aligned separately against the wheat reference genome IWGSC RefSeq v2.1 [International Wheat Genome Sequencing Consortium (IWGSC), 2018; available at: https://wheat-urgi.versailles.inra.fr/Seq-Repository/Assemblies] using BWA-MEM (version 0.7.17; Li, 2013, Preprint). Reads with a mapping quality >30, indicative of ∼99.9% confidence that the read has been placed in the correct location, were filtered and duplicate reads were removed using Sambamba (version 0.6.6, Tarasov *et al*., 2015). Subsequently, reads were annotated to high-confidence genes using the IWGSC RefSeq v2.1 gene annotation (http://wheat-urgi.versailles.inra.fr/Seq-Repository/Assemblies), employing the featureCounts option of the Subread package (version 1.6.2, Liao *et al*., 2013). Read counting was performed with a read extension to the 3’ end of 100 base pairs, in accordance with MACEseq only sequencing the 3’ end of each fragment. Multi-mapping reads were excluded from further analysis. Gene expression levels, indicated by the sequencing reads, were counted separately for each genotype, distinguishing between the ‘Intro’ and ‘Sonalika’ genotypes. An overview of sequencing depth and mapping statistics for each library is provided in Supplementary Table S1.

Reads were then normalized to counts per million, and log fold change- and false disovery rate (FDR)-corrected probability values were calculated using the edgeR package (version 3.36.0, Robinson *et al*., 2010). Specifically, a common dispersion value across all genes was estimated by maximizing the negative binomial conditional common likelihood. Tag-wise dispersion values were subsequently estimated by an empirical Bayes method based on weighted conditional maximum likelihood. Finally, gene-wise exact tests for differences in the means between the drought stress history sets were conducted, resulting in log fold change- and FDR-corrected probability scores. All three replicates for each described set of drought stress history per genotype were grouped together, allowing for their direct 1:1 comparison. Genes with a log_2_ fold change(|log_2_FC|>2) and an FDR<0.05 were classified as differentially expressed.

### Working definitions for identification of intergenerational and transgenerational transcriptional memory

In this study, we aimed to identify intergenerational and transgenerational memory genes that are regulated at the transcriptional level. In the preceding study, intergenerational stress memory was defined as stress memory that occurs when both the parental generation and the progeny generation are directly exposed to the stressor during the reproductive phase. This memory is mediated by cues introduced into the seed or embryo by the parental plant. Secondly, transgenerational stress memory was defined as stress memory observed in a generation that has not been directly exposed to the stressor but exhibits effects due to ancestral exposure during the reproductive stage. This occurs when the grandparental generation was exposed to the stress, but the parental generation experienced a recovery period without stress (Kambona *et al*., 2023a).

Applying these two definitions to transcriptional memory, for this study, we define intergenerational transcriptional memory as changes in gene expression that occur in both the parental and progeny generations as a direct result of stress exposure during the reproductive phase. An example of intergenerational transcriptional memory would be if drought stress affects gene expression in the parental generation and these gene expression changes are modified or sustained in the immediate progeny generation. Accordingly, we define transgenerational transcriptional memory as changes in gene expression observed in the progeny generation, whose parent was not directly exposed to the stressor but influenced by the exposure to stress in the grandparental generation. For example, if the grandparental generation experienced drought stress and the parental generation did not, yet the progeny still show stable altered gene expression due to the grandparental stress exposure that persists after drought recovery, then this would be an example of transgenerational transcriptional memory.

### Determination of intergenerational memory genes

In the seed transcriptome data analysis, we first conducted the initial identification of drought-responsive genes by comparing gene expressions between C1D2 and C1C2. We then examined significant responses in D1D2 versus C1D2 to see how these initial drought-responsive genes behave under continued drought stress across generations (C1D2 versus C1C2 compared with D1D2 versus D1C2). For leaf tissue transcriptomes, we compared C1C2D3 versus C1C2C3 to establish the drought-responsive genes and then analyzed responses in C1D2D3 versus C1C2D3 and D1D2D3 versus C1D2D3 (C1C2D3 versus C1C2C3 compared with C1D2D3 versus C1C2D3 compared with D1D2D3 versus C1D2D3).

Genes were categorized based on their expression patterns: increased transcript abundance (+) or decreased transcript abundance (−). For example, a gene classified as (+/+) would have higher expression levels in both comparisons: C1D2 versus C1C2 and D1D2 versus C1D2. Thus, four categories of intergenerational memory genes were defined from the seed transcriptome: (+/+), (+/−), (−/+), and (−/−). For the leaf transcriptomes, a gene in the (+/+/+) category would be elevated in all three comparisons: C1C2D3 versus C1C2C3, C1D2D3 versus C1C2D3, and D1D2D3 versus C1D2D3. Given the complexity of the analysis, we focused on eight intergenerational response patterns in the leaf transcriptome data: (+/+/+), (+/+/−), (+/−/−), (−/−/−), (−/+/+), (−/−/+), (−/+/−), and (+/−/+). In these designations, the first sign represents higher (+) or lower (−) transcript levels in C1C2D3 relative to the control (C1C2C3). The second sign represents higher (+) or lower (−) transcript levels after two stress encounters (C1D2D3) compared with one stress encounter (C1C2D3). The third sign represents higher (+) or lower (−) transcript levels after three stress encounters (D1D2D3) compared with two stress encounters (C1D2D3).

### Determination of transgenerational memory genes

It was not possible to identify transgenerational memory genes using the seed data because we lacked the critical control needed to isolate the effect of grandparental drought without the confounding influence of direct drought exposure in the parental generation. Therefore, in leaves, we identified transgenerational memory genes based on their ability to maintain altered expression patterns induced by ancestral drought stress, even when the immediate parental generation was not directly exposed to drought. Specifically, drought-induced changes in gene expression had to persist into the progeny generation despite the absence of direct drought exposure in the parental generation. The first step involved identifying genes responsive to grandparental drought stress by comparing expression fold changes between D1C2C3 (grandparental drought) and C1C2C3 (control). Next, we assessed the expression of these genes in a progeny generation without direct drought exposure in the parental generation by comparing D1C2D3 (grandparental and offspring drought) with D1C2C3 (grandparental drought only). Consequently, the analysis was conducted through a stepwise comparison: first, D1C2C3 versus C1C2C3, followed by D1C2D3 versus D1C2C3. Therefore, in general, intergenerational and transgenerational drought memory genes were identified through stepwise differential expression analysis within each genotype, targeting persistent changes across generations. Overlapping differentially expressed genes (DEGs) across these comparisons were visualized and selected using Venn diagrams generated with Venny 2.1.0. While this approach captures candidate genes for genotype-specific memory based on their presence in the unique DEG lists, it does not formally test genotype×drought history interactions. Future analyses using statistical models incorporating such interactions could provide stronger evidence for genotype-specific transcriptional memory.

### Memory gene expression patterns

To gain deeper insights into tissue-specific expression profiles, we separately examined the expression of memory genes in seeds and leaves across the different drought stress history using heatmap visualizations. For the seed transcriptome, the relative expression levels of memory genes were illustrated across the four distinct drought stress history sets: C1C2, C1D2, D1C2, and D1D2. Similarly, heatmaps for the leaf transcriptome data depicted gene expression patterns under six drought stress conditions: C1C2C3, C1C2D3, D1C2C3, D1C2D3, C1D2D3, and D1D2D3 that were used in this study. To highlight expression variability, one representative gene from each identified intergenerational response pattern in the leaf transcriptome was randomly selected, and bar plots were generated to visualize transcriptional patterns in each genotype.

### Gene Ontology enrichment analysis

GO enrichment analysis was performed under default settings using ShinyGO v0.77 on the identified intergenerational and transgenerational memory genes. All query genes were converted to ENSEMBL gene IDs or STRING-db protein IDs for analysis. Enrichment *P*-values were calculated using the hypergeometric test, and FDR correction was applied via the Benjamini–Hochberg method to account for multiple testing. Fold enrichment was calculated as the ratio of the percentage of genes in our list to that in the history genes. Pathways with an FDR<0.05 were deemed significant and ranked based on both FDR and fold enrichment. To reduce redundancy, pathways sharing 95% of their genes and 50% of the words in their names were consolidated, with only the most significant pathway retained. Given the extensive number of GO terms, results were ranked by enrichment FDR and fold enrichment. FDR *q*-values were used to adjust *P*-values for multiple testing, controlling for type I errors. Fold enrichment, indicating the magnitude of enrichment, was a key metric of effect size, with higher values signifying stronger enrichment.

### Correlation analysis between transcriptional and physio-morphological responses to repeated drought

We integrated the expression levels of the identified distinct intergenerational and transgenerational drought memory genes from the leaf transcriptome of each genotype with corresponding physiological and morphological changes under varying drought stress histories. Since these distinct memory genes were significantly enriched in pathways related to stress responses, signaling, and physiological processes, we considered them to be biologically meaningful candidates for exploring their relationships with physiological and morphological traits. Pearson correlation calculations were carried using the cor() function in R, separately for the distinct intergenerational and transgenerational memory genes in each genotype. We ranked the genes based on their correlation with the traits by calculating the mean absolute correlation and selected the top 20 genes with the highest values from each file for presentation and further discussion as they had the strongest relationships with the traits of interest. By integrating both the enrichment analysis and correlation analysis, we hoped to focus on genes with strong biological relevance, making them suitable candidates for further investigation and potentially linking them to drought stress memory mechanisms in the studied genotypes. The results were visualized using heatmaps, facilitating the identification of significant correlations.

## Results

### Physiological and morphological traits under varying drought stress history

To characterize the status of the plants under water stress conditions, several trends in physiological and morphological traits were observed across different drought stress history. The quantum yield of PSII was significantly influenced by stress history under both drought and control conditions (Table 1). Seedlings from seeds with prior drought exposure (C1D2, D1C2, and D1D2) exhibited higher photochemical quantum yield compared with control seeds (C1C2), indicating enhanced PSII efficiency (Supplementary Fig. S1A–C). Under drought conditions, the mean photochemical quantum yield for D1C2 (0.716) was 5.96% higher than for C1C2 (0.676), while D1D2 (0.711) showed a 5.06% increase compared with C1C2. Under control conditions, C1D2 (0.721), D1C2 (0.733), and D1D2 (0.703) exhibited increases of 8.39, 10.23, and 5.73%, respectively, compared with C1C2 (0.665).

**Table 1.** ANOVA significance levels for the effects of stress history (SH), genotype, and their interaction (SH×genotype) on measured traits

Treatment	Source of variation	df	Quantum yield of PSII	Stomatal conductance	Assimilation rate	ABA	Root surface area	Root crossings
Drought	SH	3	**	***	***	***	*	*
	Genotype	1	ns	**	ns	***	ns	.
	SH×genotype	3	ns	***	***	***	ns	ns
Control	SH	3	***	***	***	***	*	**
	Genotype	1	ns	ns	ns	.	*	*
	SH×genotype	3	ns	**	**	***	**	*

Each genotype (‘Intro’ and ‘Sonalika’) included four parental combinations based on prior drought stress exposure (C1C2, C1D2, D1C2, and D1D2), which were used to define the stress history (SH) factor. All traits were measured under the final treatment (drought or control), representing the last applied condition in the experimental design. ‘.’, ‘*’, ‘**’, and ‘***’ denote significance at *P*<0.1, *P*<0.05, *P*<0.01, and *P*<0.001, respectively; ns indicates non-significance.

Stomatal conductance was impacted by stress history, genotype, and their interaction (stress history×genotype) under drought conditions. Under control conditions, stress history and stress history×genotype effects remained significant, underscoring the combined influence of stress history and genotype interactions on stomatal regulation (Table 1). Under drought treatment, ‘Sonalika’ seedlings from the D1C2 seed set demonstrated the highest stomatal conductance (0.213 mol m^−2^ s^−1^), a 136% increase compared with the seedlings from the C1C2 seed set (0.090 mol m^−2^ s^−1^), while those from C1D2 and D1D2 seed sets showed increases of 76% and 101%, respectively. Conversely, ‘Intro’ seedlings from the C1D2 seed set under drought exhibited a stomal conductance of 0.176 mol m^−2^ s^−1^, a 52% increase relative to those from C1C2 (0.115 mol m^−2^ s^−1^) (Supplementary Fig. S2, Drought A). When the average of the two genotypes was considered, the seedlings with drought history (C1D2, D1C2, and D1D2) represented increases (62.4, 53.2, and 60.2%, respectively) over those with C1C2 history under drought treatment (Supplementary Fig. S2, Drought B). Under control conditions, ‘Sonalika’ and ‘Intro’ seedlings from C1C2 seed sets showed higher stomatal conductance, whereas those from C1D2 seed sets exhibited reductions of 57% in ‘Sonalika’, while in ‘Intro’ seedlings from D1D2 seed sets showed decreases of 46% (Supplementary Fig. S2, Control C). Generally, under control conditions, the seedlings with C1D2 and D1D2 history displayed marked reductions (41% and 40%, respectively) compared with the seedlings from C1C2 (Supplementary Fig. S2, Control D).

Assimilation rate was consistently influenced by stress history and stress history×genotype interaction under both treatments (Table 1). Under drought conditions, seedlings from the C1C2 seed set exhibited the lowest assimilation rates for ‘Sonalika’ (4.58 μmol m^−2^ s^−1^), while seedling from C1D2 (6.90 μmol m^−2^ s^−1^), D1C2 (8.58 μmol m^−2^ s^−1^), and D1D2 (8.26 μmol m^−2^ s^−1^) seed sets demonstrated a higher rate, representing 51, 87, and 80% increases, respectively, relative to C1C2 (Supplementary Fig. S3, Drought A). When examining combined genotypes, assimilation rates under drought conditions were generally higher in all seedlings from seed sets with drought stress history (Supplementary Fig. S3, Drought B). Under control conditions, ‘Intro’ seedlings from the C1C2 seed set had the highest assimilation rate (7.35 μmol m^−2^ s^−1^), close to those of C1D2 (6.35 μmol m^−2^ d^−1^) and D1C2 (7.19 μmol m^−2^ s^−1^) seedlings, but those from the D1D2 seed set (4.42 μmol m^−2^ s^−1^) exhibited the lowest assimilation rate, showing a 40% reduction relative to C1C2 (Supplementary Fig. S3, Control C). Compared with C1C2 seedlings, the D1D2 seed set seedlings exhibited a reduction of 21% under control conditions (Supplementary Fig. S3, Control D).

ABA levels were significantly affected by stress history and stress history×genotype interaction in both treatments, with additional genotype-specific variation under drought conditions (Table 1). Under drought conditions, ‘Sonalika’ seedlings from drought-experienced seed sets showed significant reductions in ABA content, with C1D2, D1C2, and D1D2 exhibiting decreases of 87.1, 97.1, and 94.7%, respectively, compared with C1C2. ‘Intro’ seedlings from the D1C2 seed set recorded the highest ABA levels (0.493 μg ml^–1^), representing a 196% increase compared with C1C2 (0.167 μg ml^–1^) (Supplementary Fig. S4, Drought A). When considering combined genotype means, seedlings from the C1C2 history had the highest ABA levels under drought conditions, while those from stressed history had lower levels (Supplementary Fig. S4, Drought B). Under control conditions, ‘Sonalika’ seedlings from the C1D2 seed set displayed the highest levels (0.877 μg ml^–1^), marking a dramatic 1362% increase relative to C1C2 (0.06 μg ml^–1^). Similarly, ‘Intro’ seedlings from the D1D2 seed set showed the highest ABA content (0.67 μg ml^–1^), a 617% increase compared with C1C2 (0.093 μg ml^–1^). (Supplementary Fig. S4, Control B). When analyzing the combined genotype means, seedlings from the C1D2 and D1D2 history exhibited higher ABA levels compared with those from the C1C2 history. Notably, the C1D2 seedlings had the highest ABA levels (0.565 μg ml^–1^), representing a remarkable 637% increase compared with C1C2 seedlings (0.077 μg ml^–1^) (Supplementary Fig. S4, Control D).

Root morphological traits also displayed notable responses to stress history. Under 20% PEG, root surface area was significantly influenced by stress history (*P*<0.05). In contrast, under 0% PEG, stress history (*P*<0.05), genotype (*P*<0.05), and stress history×genotype interaction (*P*<0.01) all had significant effects (Table 1). Under drought conditions, the combined genotype means revealed the largest root surface area in seedlings from the D1C2 seed set (63.56 cm^2^), a 45% increase over the C1C2 history (43.94 cm^2^), while the D1D2 history also showed a substantial 44% increase (63.08 cm^2^) (Supplementary Fig. S5, 20% PEG A). Under control conditions, ‘Sonalika’ seedlings from the D1D2 seed set exhibited the largest root surface area (71.65 cm^2^), an 86% increase over the C1C2 seed set (38.42 cm^2^), followed by the D1C2 seed set (67.05 cm^2^), which showed a 74% increase compared with C1C2 (Supplementary Fig. S5, 0% PEG B). Generally, seedlings from the D1C2 history had the largest root surface area (62.86 cm^2^), representing an 87% increase compared with C1C2 (33.63 cm^2^) (Supplementary Fig. S5, 0% PEG C).

Stress history had a significant effect on root crossings under 20% PEG (*P*<0.05), while under 0% PEG, stress history (*P*<0.01), genotype (*P*<0.05), and stress history×genotype interaction (*P*<0.05) were all significant (Table 1). When considering combined genotype means, the highest root crossing density under drought conditions was observed in the D1D2 seed set (636 crossings), representing a 65% increase compared with the C1C2 seed set (386 crossings) (Supplementary Fig. S6, 20% PEG A). Under control conditions, ‘Sonalika’ seedlings from the D1C2 seed set exhibited the highest root crossing density (499 crossings), a 151% increase over C1C2 (199 crossings), followed by the D1D2 seed set with a 129% increase (456 crossings) (Supplementary Fig. S6, 0% PEG B). Combined, the D1C2 seed set displayed the highest root crossing density (446 crossings), representing a 172% increase compared with the C1C2 seed set (164 crossings) (Supplementary Fig. S6, 0% PEG C).

### Intergenerational drought memory genes

To gain insight into the transcriptional signatures of intergenerational drought stress memory, we identified genes in both seed and leaf transcriptomes of ‘Intro’ and ‘Sonalika’ that showed altered expression in response to recurrent drought exposure across generations. From the seed transcriptome, 472 genes in ‘Intro’ and 478 genes in ‘Sonalika’ significantly increased or decreased their transcript levels during the first drought stress encounter (C1D2 versus C1C2). Specifically, 287 and 247 genes were elevated while 185 and 231 genes were reduced in ‘Intro’ and ‘Sonalika’, respectively. These genes were considered as potential drought-responsive candidates. In ‘Intro’, 341 distinct genes were identified, with 186 showing higher expression levels and 155 showing lower expression levels, while ‘Sonalika’ exhibited 347 distinct genes, with 162 elevated and 185 reduced. There were 131 common genes in both genotypes, of which 81 genes were increased, while 26 genes were decreased in both genotypes. We also observed that 20 genes high in ‘Intro’ were reduced in ‘Sonalika’, while four genes with low transcript abudance in ‘Intro’ were elevated in ‘Sonalika’. Among these drought-responsive genes, we identified 12 (+/+), 140 (+/−), and 77 (−/+) genes in ‘Intro’. In ‘Sonalika’, we identified 37 (+/−) and 20 (−/+) genes (Table 2). Of the total 229 intergenerational memory genes in ‘Intro’ and 57 in ‘Sonalika’, 166 and 43 genes, respectively, were among the identified distinct drought-responsive genes.

**Table 2.** Drought-responsive, intergenerational memory, and transgenerational memory genes identified in wheat seed and leaf transcriptomes

Gene category	Seed transcriptome (intergenerational memory genes)		Leaf transcriptome (intergenerational memory genes)		Leaf transcriptome (transgenerational memory genes)	
	‘Intro’	‘Sonalika’	‘Intro’	‘Sonalika’	‘Intro’	‘Sonalika’
Drought-responsive genes	472	478	5398	5191	1706	4875
Memory genes (total)	229	57	119	287	500	3029
Memory gene patterns						
(+/+)	12	0	−	—	39	166
(−/−)	0	0	—	—	17	103
(+/−)	140	37	—	—	328	1863
(−/+)	77	20	—	—	116	879
(+/+/+)	—	—	1	0		
(−/−/−)	—	—	1	2		
(+/−/−)	—	—	35	22		
(+/+/−)	—	—	1	26	—	—
(−/−/+)	—	—	13	28	—	—
(−/+/+)	—	—	37	21	—	—
(−/+/−)	—	—	8	80	—	—
(+/−/+)	—	—	23	108	—	—

Drought-responsive genes were identified to serve as the foundation for identifying intergenerational and transgenerational memory genes: in seeds (C1D2 versus C1C2) and in leaves (C1C2D3 versus C1C2C3). Intergenerational memory genes were further assessed from these by comparing D1D2 versus D1C2 (seeds) and C1D2D3 versus C1C2D3 and D1D2D3 versus C1D2D3 (leaves). Transgenerational memory genes were identified from genes responsive to grandparental drought (D1C2C3 versus C1C2C3) that maintained altered expression in the progeny (D1C2D3 versus D1C2C3). C and D indicate drought and control conditions, respectively, and numbers following them indicate the generations; 1=grandparental, 2=parental and 3=offspring. Genes listed in this table were identified as differentially expressed using an FDR <0.05 and |log_2_ fold change|≥2.

From the leaf transcriptome, we found that 5398 and 5192 genes in ‘Intro’ and ‘Sonalika’, respectively, significantly increased or decreased their transcript levels during the first drought stress encounter (C1C2D3 versus C1C2C3). Specifically, 2272 and 2274 genes were increased, and 3126 and 2917 genes were decreased in ‘Intro’ and ‘Sonalika’, respectively. In ‘Intro’, we identified 3534 distinct genes, with 1502 raised and 2032 lowered transcript levels. In ‘Sonalika’, 3327 distinct genes were identified, comprising 1511 raised and 1816 lowered transcript abundance. Across both genotypes, 1864 genes were common, of which 674 had elevated and 1005 had reduced expression. In addition, 96 genes with high expression levels in ‘Intro’ were lowered in ‘Sonalika’, while 89 genes whose expression levels were reduced in ‘Intro’ were raised in ‘Sonalika’. From these drought-responsive genes, in ‘Intro’, we identified one (+/+/+), one (−/−/−), 35 (+/−/−), one (+/+/−), 13 (−/−/+), 37 (−/+/+), eight (−/+/−), and 23 (+/−/+) genes. In ‘Sonalika’, we identified two (−/−/−), 22 (+/−/−), 26 (+/+/−), 28 (−/−/+), 21 (−/+/+), 80 (−/+/−), and 108 (+/−/+) genes (Table 2). Of the total 119 and 287 intergenerational memory genes identified in ‘Intro’ and ‘Sonalika’, respectively, 53 genes in ‘Intro’ and 131 genes in ‘Sonalika’ were among the identified distinct drought-responsive genes.

### Transgenerational drought memory genes

To gain insight into the transcriptional signatures of transgenerational drought stress memory, we identified genes in ‘Intro’ and ‘Sonalika’ that were differentially expressed in the progeny, indicating persistent transcriptional effects of ancestral stress despite recovery in the parental generation. In ‘Intro’, 1706 genes, and in ‘Sonalika’, 4875 genes were significantly differentially expressed when D1C2C3 was compared with C1C2C3, indicating their responsiveness to grandparental drought stress. Among these, 1193 genes were elevated and 513 were reduced in ‘Intro’, while 2940 genes were high and 1935 showed reduced expression levels in ‘Sonalika’. A total of 547 genes were common between the two genotypes, with 1159 genes distinct to ‘Intro’ and 4328 genes distinct to ‘Sonalika’. Among the common genes, expression of 50 was raised in ‘Intro’ but reduced in ‘Sonalika’, while 31 genes were expressed at a low level in ‘Intro’ but raised in ‘Sonalika’. Among these grandparental drought-responsive genes, we identified 39 and 166 (+/+), 328 and 1863 (+/−), 17 and 103 (−/−), and 116 and 897 (−/+) genes in ‘Intro’ and ‘Sonalika’, respectively (Table 2). Notably, 224 and 2613 transgenerational memory genes in ‘Intro’ and ‘Sonalika’, respectively, were also part of the set of distinct grandparental drought-responsive genes.

### Gene expression patterns of drought stress memory genes

**Fig. 2.**
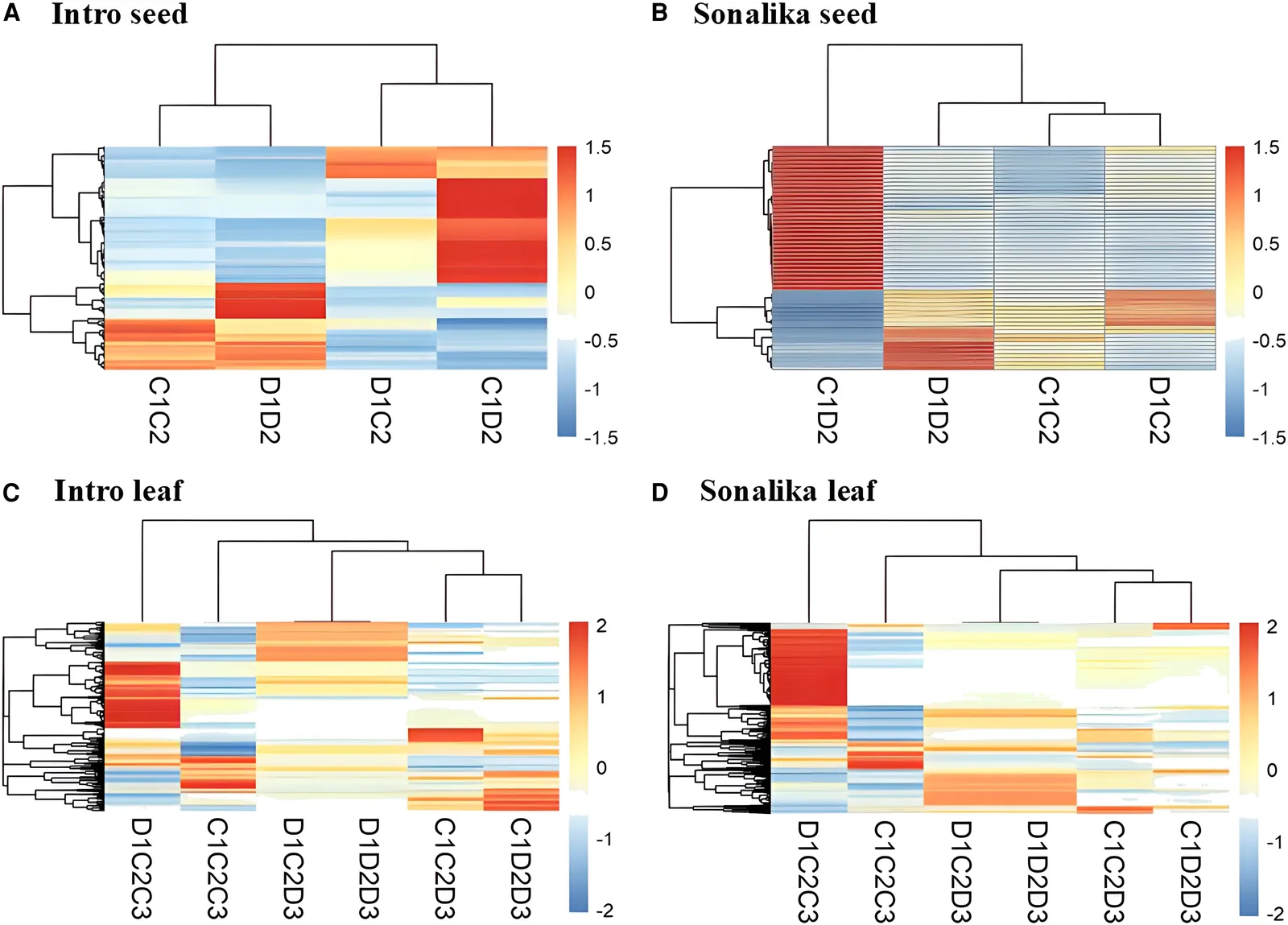
Heatmap visualization of memory gene expression patterns in response to drought stress across different history and genotypes. (A and B) Heatmap of seed transcriptome data showing the expression levels of memory genes across four types of drought stress history in (A) ‘Intro’ and (B) Sonalika’. (C and D) Heatmap of leaf transcriptome data depicting the expression levels of memory genes under six types of drought stress history in (C) ‘Intro’ and (D) ‘Sonalika’. Each heatmap illustrates the relative expression levels of memory genes, with rows representing individual genes and columns representing specific stress history. Color intensity represents relative gene expression levels, as indicated by the scale bars shown alongside each heatmap. Differential expression was determined using an FDR<0.05 and |log_2_ fold change|≥2.

To obtain information on the genes that respond specifically across different drought stress history, we analyzed gene expression profiles separately for seed and leaf tissues in each genotype using heatmap visualizations. In both seed and leaf transcriptomes, memory genes displayed condition-specific increases or decreases in transcript abundance. In seeds, a subset of genes showed higher transcript abundance in D1D2 but consistently lower abundance in D1C2 and C1D2 in ‘Intro’, whereas in ‘Sonalika’, a subset of genes displayed consistently higher transcript abundance across all drought stress scenarios. In both genotypes, other genes showed significant variability depending on the stress history (Fig. 2A, B). In the leaf transcriptome, expression profiles under the six sets of drought stress history demonstrated greater complexity. Notably, genes in the C1C2C3 condition exhibited milder expression changes compared with the pronounced shifts observed in D1D2D3 (Fig. 2C, D).

**Fig. 3.**
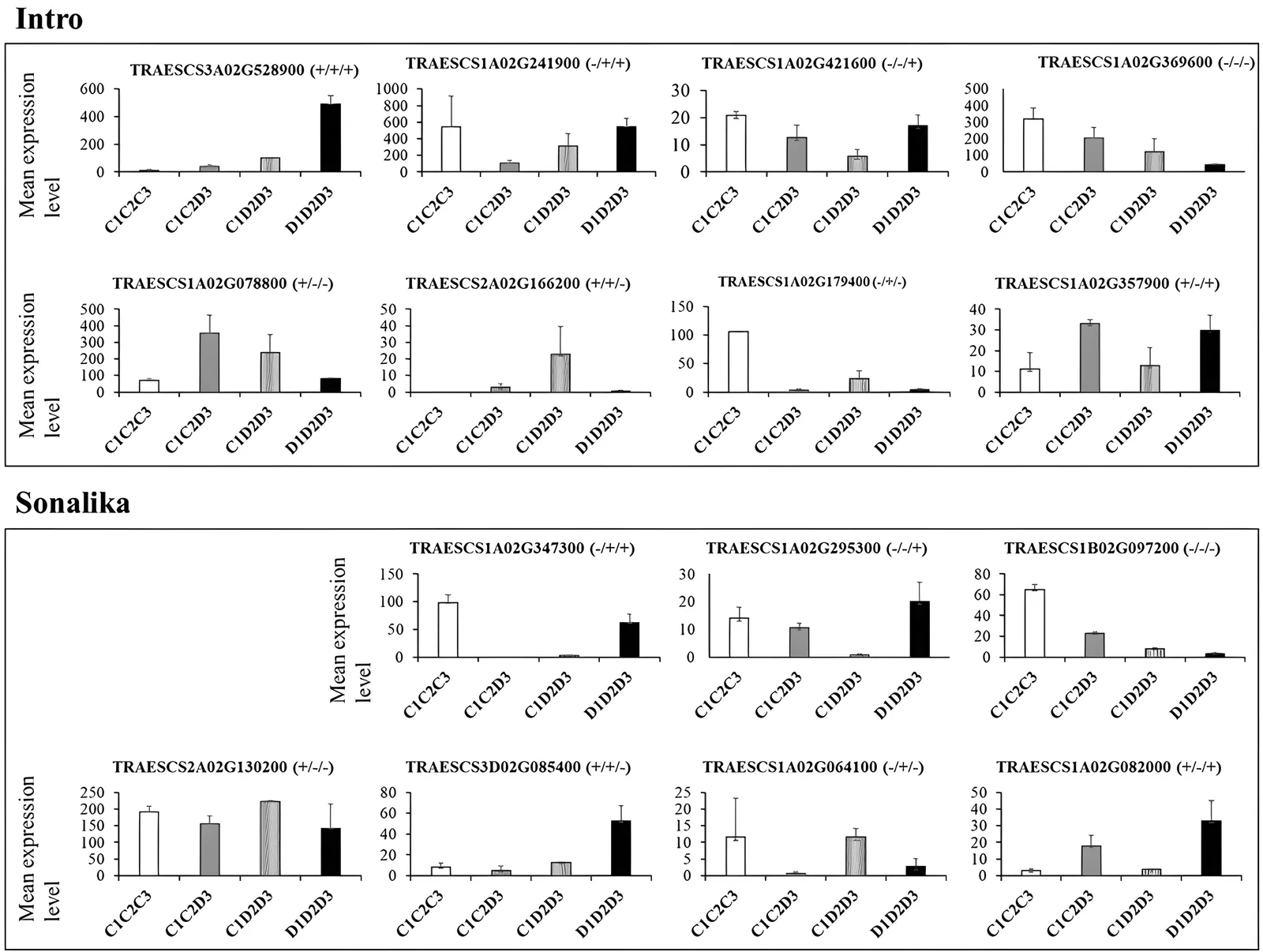
Expression patterns of intergenerational drought stress memory genes in leaves for ‘Intro’ and ‘Sonalika’ genotypes. Each bar represents the mean expression level across three replicates, with error bars indicating the SE. The *x*-axis shows different drought stress history: C1C2C3 (control), C1C2D3 (offspring drought stress), C1D2D3 (combined parental and offspring drought stress), and D1D2D3 (multigenerational drought stress). The *y*-axis represents the normalized expression levels. Gene categories comprise (+/+/+), (−/+/+), (−/−/+), (−/−/−), (+/−/−), (+/+/−), (−/+/−), and (+/−/+), with one representative gene from each category, which was randomly selected. In these designations, the first sign indicates higher (+) or lower (−) transcript levels in C1C2D3 relative to the control (C1C2C3). The second sign reflects higher (+) or lower (−) transcript levels after two stress encounters (C1D2D3) compared with one stress encounter (C1C2D3). The third sign represents higher (+) or lower (−) transcript levels after three stress encounters (D1D2D3) relative to two stress encounters (C1D2D3). These genes were classified as differentially expressed if they showed |log_2_ fold change|>2 and FDR<0.05.

Bar plots of representative genes from each intergenerational response pattern further validated these findings, illustrating distinct transcriptional dynamics under different conditions. In both genotypes, genes associated with the (+/+/+) pattern showed consistent up-regulation across all stress history, while genes in the (+/+/−) pattern displayed a marked reduction in expression under D1D2D3 compared with C1D2D3 (Fig. 3).

### Enrichment analysis of the identified intergenerational drought memory genes in the seed transcriptome

**Fig. 4.**
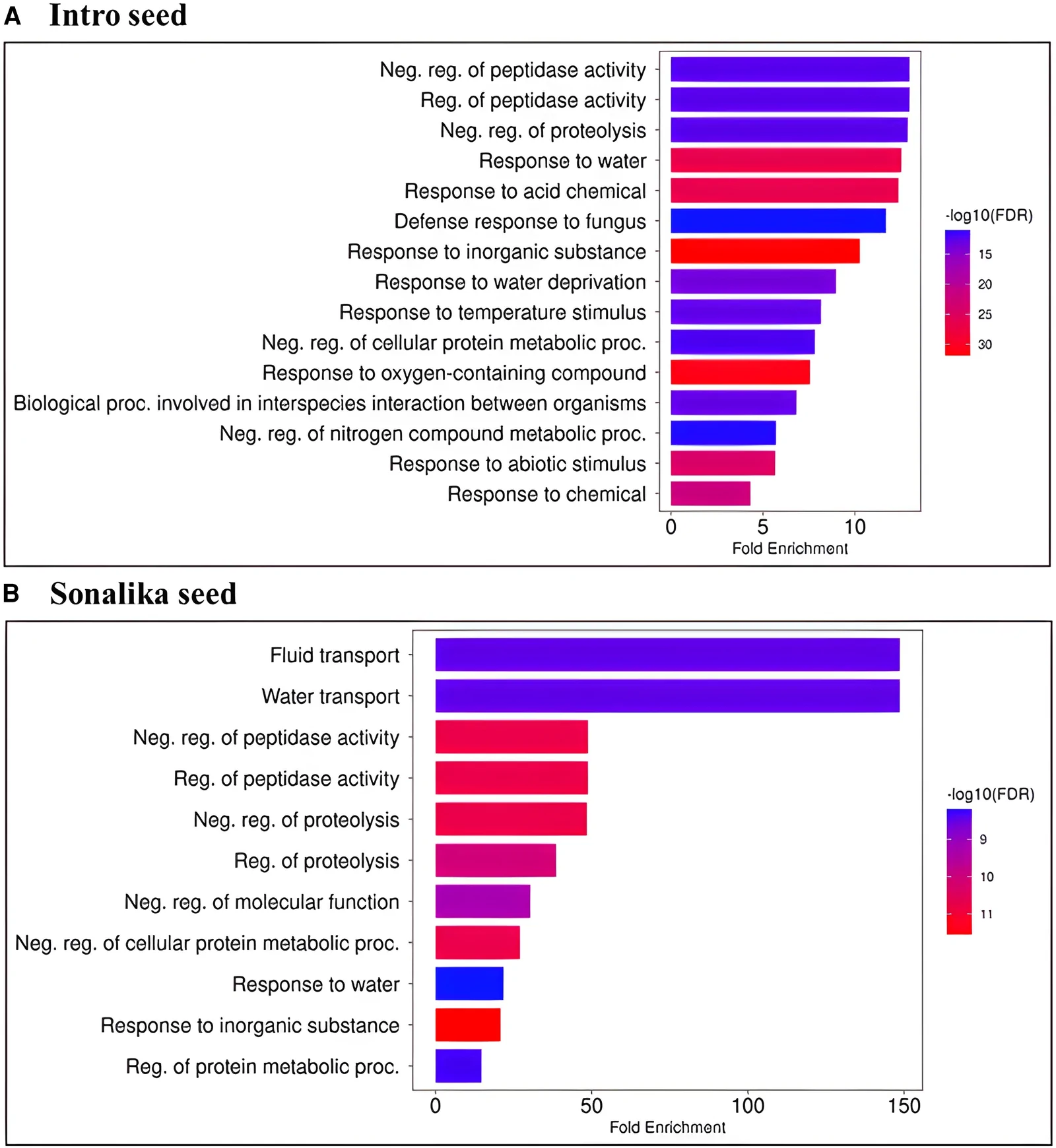
Top enriched GO terms in intergenerational drought stress memory genes of the seed transcriptome. (A) ‘Intro’ and (B) ‘Sonalika’. The *y*-axis shows the most significantly enriched Gene Ontology (GO) terms related to drought stress memory, with each term depicted as a bar. The length of each bar reflects the fold enrichment of the GO term, while the color gradient represents statistical significance, as indicated by the –log10(FDR) value. Longer bars denote higher fold enrichment and variations in shading intensity show differences in statistical significance, highlighting key pathways involved in drought stress response and memory.

To gain insights into the biological processes and functional pathways associated with intergenerational drought memory genes with the strongest expression levels from the seed transcriptome, we performed GO enrichment analysis. Several significantly enriched terms were identified from the ‘Intro’ seed transcriptome (Fig. 4A). Key biological processes included nutrient reservoir activity, response to water, response to water deprivation, response to inorganic substances, response to oxygen-containing compounds, and seed development. Notably, pathways related to peptidase and endopeptidase activities, such as the negative regulation of peptidase activity, showed high enrichment. Processes involved in transcriptional and translational regulation, including regulation of DNA-templated transcription and translation termination, were also prominent. The analysis also highlighted the enrichment of defense response pathways, such as regulation of defense response and response to organic substances. Similarly, pathways related to oxidative stress management, including cellular response to toxic substances, cellular detoxification, and response to oxygen-containing compounds, were significantly enriched. Notably, there was enrichment of terms associated with epigenetic and structural regulation, such as DNA packaging, chromatin remodeling, histone H3K36 trimethylation, and regulation of DNA-templated transcription and termination.

Among the most enriched terms in the seed transcriptome of ‘Sonalika’ were those related to the negative regulation of proteolysis, peptidase regulator activity, and peptidase activity (Fig. 4B). Additionally, there was enrichment of processes associated with water transport, such as water channel activity and water transmembrane transporter activity. Terms related to seed viability and post-drought germination, including seed development, nutrient reservoir activity, and root system development, were prominently represented. Molecular functions crucial for managing protein degradation and prolonged stress adaptation were indicated by the enrichment of pathways such as endopeptidase inhibitors and peptidase regulator activities. Similarly, functional terms related to oxidative stress responses, including amylase activity, superoxide dismutase (SOD) activity, and catalase (CAT) activity, were highly enriched. Pathways related to epigenetic regulation showed significant enrichment, particularly those associated with acetyltransferase complexes, such as protein acetyltransferase complex, protein acetylation, histone H3K9 acetylation, and histone H3K14 acetylation.

### Enrichment analysis of the identified transgenerational and intergenerational drought memory genes in the leaf transcriptome

**Fig. 5.**
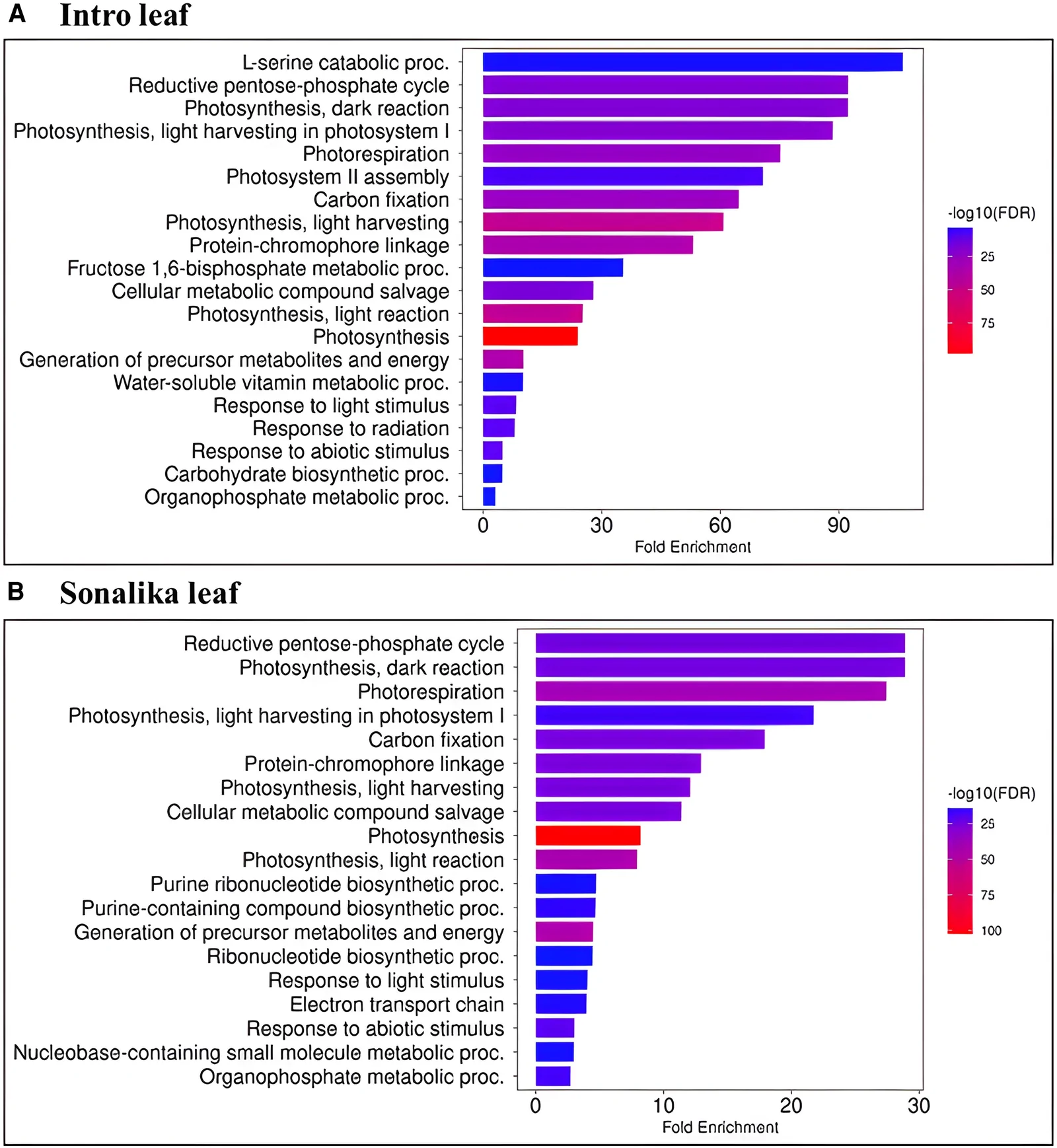
Top enriched GO terms in transgenerational and intergenerational drought stress memory genes of the leaf transcriptome. (A) ‘Intro’ and (B) ‘Sonalika’. The *y*-axis shows the most significantly enriched Gene Ontology (GO) terms related to drought stress memory, with each term depicted as a bar. The length of each bar reflects the fold enrichment of the GO term, while the color gradient represents statistical significance, as indicated by the –log10(FDR) value. Longer bars denote higher fold enrichment, and differences in shading intensity indicate variation in statistical significance, highlighting key pathways involved in drought stress response and memory.

To better understand the functional roles of the identified intergenerational and transgenerational drought memory genes from the leaf transcriptome, we conducted enrichment analysis. The analysis of the leaf transcriptome of the ‘Intro’ genotype revealed exceptionally high fold enrichment in pathways associated with photosynthesis, including PSI, the photosynthetic electron transport chain, the cytochrome *b*_6_*f* complex, chlorophyll binding, and the photosynthetic membrane. Similarly, pathways related to thylakoid and chloroplast structures, such as the thylakoid, plastid thylakoid membrane, and chloroplast thylakoid membrane, were significantly enriched. Processes involved in light harvesting and carbon assimilation, including PSII and carbon fixation pathways, were also prominently enriched. Additionally, the analysis identified pathways linked to stress responses and secondary metabolic processes, such as the response to light stimulus. Pathways related to ion and metal homeostasis, particularly cellular metal ion homeostasis and cellular potassium ion homeostasis, were enriched. Notable enrichment was observed in stress-related pathways, including the response to hydrogen peroxide and cellular response to chemical stress. Other notable terms included isoprenoid metabolic process and lipid biosynthetic process (Fig. 5A).

In the ‘Sonalika’ genotype, the analysis also revealed significant enrichment in photosynthesis-related pathways, including key terms such as thylakoid, photosynthesis, photosynthetic membrane, PSI, and Rubisco activity. Additionally, pathways related to structural and functional maintenance of plastids, such as plastid envelope and plastid membrane, were enriched. Pathways related to protein transport and localization, including intracellular protein transmembrane transport and protein targeting to the endoplasmic reticulum (ER), exhibited high fold enrichment. The analysis also emphasizes the importance of metabolic processes, with notable enrichments in pathways such as chloroplast rRNA processing and starch catabolic process. Other enriched key terms included response to osmotic stress, cellular response to stress, response to reactive oxygen species (ROS), cellular response to lipids, and positive regulation of signal transduction. Moreover, specific enzymatic activities, such as ferric iron binding and glycerol-3-phosphate *o*-acyltransferase activity, showed high fold enrichment (Fig. 5B).

### Correlation between distinct memory gene expression levels for each genotype and morpho-physiological traits

**Fig. 6.**
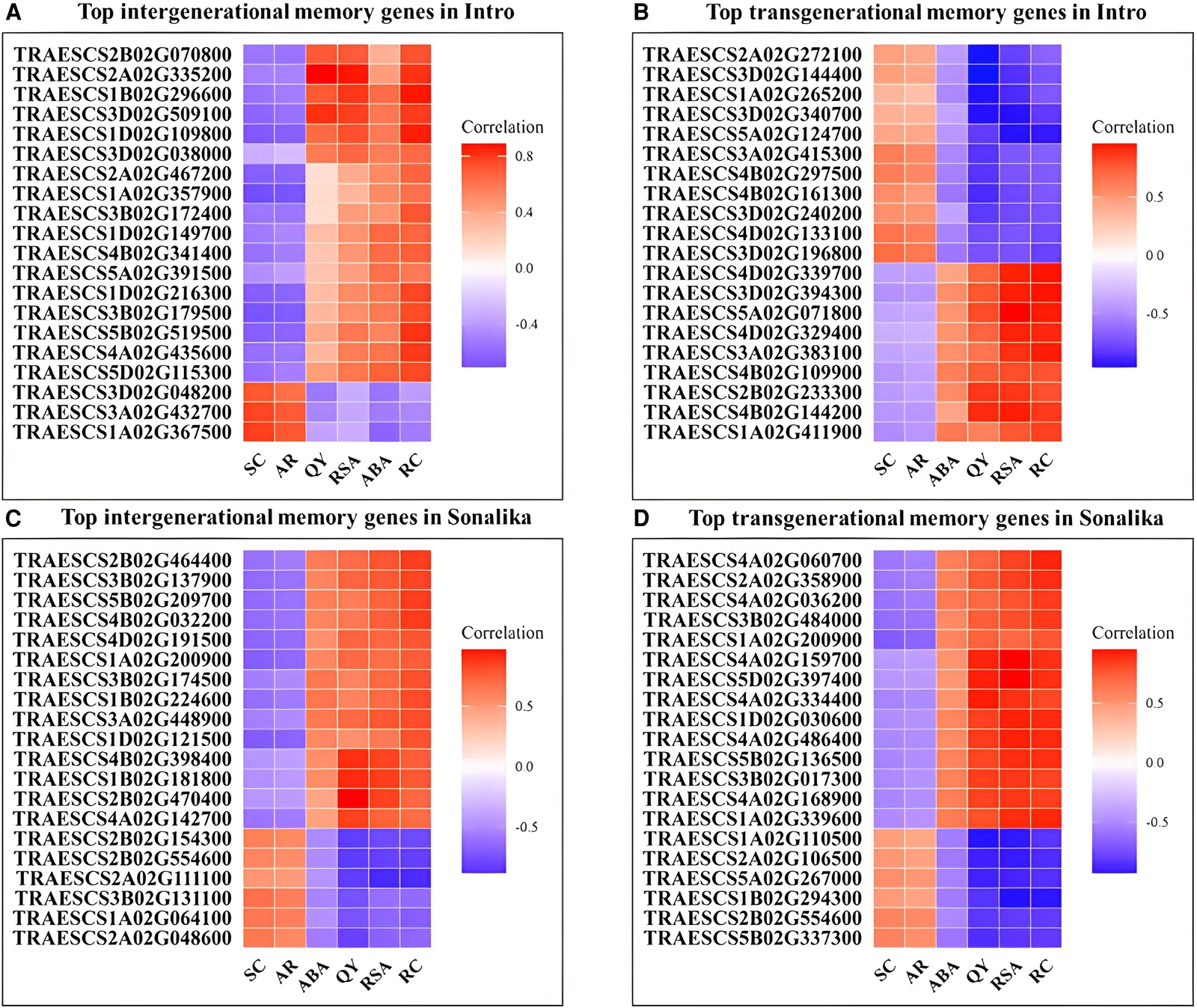
Heatmap of correlations between the top 20 identified distinct intergenerational and transgenerational drought memory genes. (A) Top intergenerational memory genes in ‘Intro’, (B) Top transgenerational memory genes in ‘Intro’, (C) Top intergenerational memory genes in ‘Sonalika’, and (D) Top transgenerational memory genes in ‘Sonalika’. The genes were selected based on their mean absolute correlation values, and six physiological/morphological traits are represented: photochemical quantum yield of PSII (QY), stomatal conductance (SC), assimilation rate (AR), abscisic acid (ABA), root surface area (RSA), and root crossing (RC). The clustering of rows and columns reveals patterns of gene–trait associations. Shading intensity represents the strength and direction of the correlations according to the scale provided, with stronger positive and negative correlations indicated by increasing intensity, while values near zero indicate weak or no linear relationship between gene expression and the measured traits.

To determine whether the identified distinct memory genes in each genotype are functionally linked to drought adaptation, we analyzed the correlation between their expression levels and key morpho-physiological traits within each genotype. In both ‘Intro’ and ‘Sonalika’ genotypes, significant correlations were observed. The clustering of rows and columns in the heatmap grouped genes and traits with similar correlation patterns, helping to identify clusters of genes that collectively influence specific traits or groups of traits that are similarly affected by gene expression changes (Fig. 6). The expression level of some genes showed a strong positive correlation with the traits (Fig. 6, red shades), suggesting that as the expression level of a gene increases, the value of the correlated trait also increases. Conversely, some genes were negatively correlated with the traits (Fig. 6, blue shades), indicating an inhibitory relationship where the expression level of a gene increases while the trait value decreases.

In both genotypes, stomatal conductance and assimilation rate formed a distinct cluster, while ABA concentration, quantum yield of PSII, and root traits grouped together in a separate cluster. In addition, genes that were negatively correlated with stomatal conductance and assimilation rate were positively correlated with ABA concentration, quantum yield of PSII, and root traits. On the other hand, genes that were positively correlated with stomatal conductance and assimilation rate were negatively correlated with ABA concentration, quantum yield of PSII, and root traits (Fig. 6A–D). Generally, the most highly correlated distinct intergenerational memory genes showed negative correlations with stomatal conductance and assimilation rate, but positive correlations with the other traits. These patterns, along with the classification of genes by their drought memory response patterns, are visualized in the clustered correlation heatmaps (Supplementary Figs S7–S10), that highlight coherent clusters of genes that may jointly influence key drought adaptation traits. The complete correlation matrix of all distinct intergenerational and transgenerational drought memory genes and traits is provided in Supplementary Dataset S1. We also provide a detailed list of the top-ranked intergenerational and transgenerational drought memory genes for each genotype, including their alternative names (if any), predicted functions, and Arabidopsis orthologs where available, in Supplementary Table S2.

## Discussion

The integration of multigenerational morpho-physiological and molecular data allowed us to uncover some of the complex adaptive mechanisms that the ‘Intro’ and ‘Sonalika’ genotypes, differing in drought tolerance, employ to respond to drought over multiple generations of varying exposure.

### Genotype-specific transcriptional regulation and putative epigenetic involvement

Analysis of the seed transcriptome revealed central differences between the two genotypes in transcripts encoding histone-modifying enzymes. While epigenetic priming is often linked to histone methylation (Liu *et al*., 2023), in our study ‘Intro’ showed enrichment of transcripts associated with H3K36me3 and chromatin remodeling, indicating a potential mechanism for transcriptional priming, though direct histone modifications were not measured. According to M.H. Yu *et al*. (2025) and Yuan *et al*. (2025), H3K36me3 is crucial for activating genes involved in light and sucrose responses, enhancing photosynthesis and metabolism, and aiding plant adaptation to environmental changes. Additionally, by analogy with pathogen memory described for SDG8-mediated H3K36me3, it is plausible that a similar mechanism enables drought stress memory (Kang *et al*., 2022). These marks are central to the formation of ‘poised’ chromatin states, in which genes are marked for potential rapid activation, yet transcriptionally silent until the appropriate stimulus is present (Ding *et al*., 2012, 2013; Yang *et al*., 2014; Lämke and Bäurle, 2017). The presence of terms such as regulation of transcription initiation/elongation/termination implies that ‘Intro’ fine-tunes gene expression kinetically, optimizing when and how strongly certain genes are expressed later when exposed to stress (Lämke and Bäurle, 2017). Furthermore, the enrichment of chromatin assembly/disassembly and nucleosome organization pathways in ‘Intro’ suggests that it actively adjusts chromatin accessibility during and after drought, helping to establish a controlled transcriptional state for key genes (Kim *et al*., 2008; Lämke and Bäurle, 2017).

In contrast, the seed transcriptome of the drought-sensitive ‘Sonalika’ genotype showed enrichment for transcripts linked to histone acetylation, particularly those associated with H3K9ac and H3K14ac, which are generally associated with an open chromatin state and rapid transcriptional activation (Kim *et al*., 2009; Wang *et al*., 2015; Lämke and Bäurle, 2017). Genes encoding acetyltransferase complexes, including SAGA (Spt-Ada-Gcn5 Acetyltransferase) and SLIK (SAGA-Like) were also enriched, highlighting the prominence of acetylation-driven chromatin remodeling. The prevalence of genes associated with H3K9ac and H3K14ac, without cDNA or histone methylation, suggests that chromatin opening in ‘Sonalika’ may lack fine-tuning or repression, potentially leading to transient, non-specific gene expression bursts during subsequent stress events to rapidly activate stress-responsive genes. Moreover, the genotype shows limited enrichment in pathways that actively restructure chromatin, implying that future transcriptional responses may rely more on reactive post-transcriptional mechanisms (Crisp *et al*., 2016). This contrasts with drought-tolerant genotypes, which often show coordinated methylation and acetylation changes that prime specific stress-responsive genes, resulting in more controlled and sustained transcriptional responses. Although less stable than methylation, histone acetylation can facilitate stress adaptation by creating a permissive chromatin environment for transcription factors and by acting as a platform for other chromatin remodelers or small RNA-mediated regulatory pathways (Kim *et al*., 2009; D’Urso and Brickner, 2014; Iwasaki and Paszkowski, 2014). This mechanism may explain the broad, reactive transcriptional program observed in ‘Sonalika’, despite the lack of stable methylation-based priming. Importantly, although initially drought sensitive, ‘Sonalika’ retains the capacity for stress memory. The acetylation-driven chromatin accessibility enables rapid activation of stress-responsive genes upon repeated drought exposure, allowing the genotype to achieve functional acclimation despite its initial vulnerability. Such a strategy may allow the genotype to quickly respond to unpredictable drought events, even if the response is less targeted. In other systems, persistent H3K9 and H3K14 acetylation has been linked to transcriptional memory, for example in heat stress and pathogen responses, suggesting that ‘Sonalika’ may employ a distinct epigenetic strategy to maintain transcriptional responsiveness across repeated stress episodes (Kim *et al*., 2012; Lämke *et al*., 2016). Nevertheless, despite its reversibility, acetylation may contribute to intergenerational or transgenerational drought acclimation by promoting rapid transcriptional reprogramming and maintaining a flexible, stress-responsive chromatin landscape. Understanding these acetylation-driven dynamics may inform breeding strategies, as transient but broad stress responsiveness could be leveraged to enhance adaptability in variable drought conditions, potentially in combination with stable epigenetic marks to achieve both flexibility and specificity in stress memory.

While our transcriptome-based enrichment analysis highlights distinct histone modification signatures associated with drought memory in these two genotypes, they both displayed evidence of intergenerational and transgenerational stress memory, showing that multiple regulatory strategies can achieve functionally similar drought acclimation (Table 2). The study of Lämke and Bäurle (2017) highlights that genotypes lacking stable heritable epigenetic modifications are still capable of mounting robust stress responses by employing broad transcriptional reprogramming. This plasticity in gene expression allows the plant to activate a wide array of stress-responsive genes dynamically, without the need for pre-established epigenetic memory. Such transcriptional activity can be transient yet sufficient for effective tolerance, underscoring the importance of flexible gene regulation beyond heritable epigenetic states. Further investigation using direct chromatin profiling will be required to elucidate the precise molecular mechanisms underlying these differences.

### Differential regulation of drought memory genes

Analysis of the intergenerational memory of drought stress revealed disparity in the number of memory genes in each genotype, suggesting that the drought memory response is specific to genetic makeup (Ramírez *et al*., 2015; Schulze *et al*., 2021). ‘Intro’ had more intergenerational drought memory genes in seeds, signifying a strategy focused on early-stage preparedness. This observation aligns with the enrichment of genes linked to histone methylation in its seed transcriptome, reflecting a stable transcriptional and epigenetic baseline set before germination, helping conserve energy by minimizing future unnecessary transcription (Fleta-Soriano and Munné-Bosch, 2016; Hilker *et al*., 2016; Lämke and Bäurle, 2017).

In the leaf transcriptome, we identified a total of 5398 and 5191 DEGs in ‘Intro’ and ‘Sonalika’, respectively, representing ∼10% of all detectable genes and highlighting the extensive impact of drought stress on gene expression within the transcriptional regulatory network of wheat. We observed that the drought-sensitive genotype ‘Sonalika’ exhibited a greater number of DEGs during the second drought exposure Table 2. This pattern has been reported across various species under prolonged stress conditions, including drought (Min *et al*., 2016), osmotic (Guo *et al*., 2017), salt (Chen *et al*., 2022), and high-temperature stress (Wang *et al*., 2018), where sensitive genotypes tend to show increased transcriptional activity as stress persists. Interestingly, ‘Sonalika’ exhibited a greater number of both intergenerational and transgenerational drought memory genes in the leaves, suggesting a more reactive and transcriptionally dynamic post-embryonic state. This observation aligns with the enrichment of genes linked to histone acetylation in its seed transcriptome, which may serve as initiating epigenetic signals that prime transcriptional networks or small RNA pathways, contributing to persistent gene activity in somatic tissues (D’Urso and Brickner, 2014; Iwasaki and Paszkowski, 2014; Amini *et al*., 2023).

### Shared drought response mechanisms in the leaf transcriptome

The analysis of the leaf transcriptome showed that some transgenerational drought memory genes exhibiting a (+/−) response pattern were shared by both genotypes, suggesting the existence of a core set of drought-responsive genes that are likely to represent universal mechanisms of stress adaptation (Bray, 2004; Guo *et al*., 2009). These genes were associated with GO terms related to photosynthesis, such as Rubisco activity, carbon fixation, photorespiration, PSII, regulation of photosynthesis, photosynthetic electron transport chain, and PSI reaction center, in both genotypes (Fig. 5). The initial up-regulation of these genes infers that those plants boost their photosynthetic capacity to maximize energy production during drought, consistent with the notion that enhanced photosynthetic activity is a common early stress response (Zhu, 2016). The subsequent decrease in transcript abundance for these genes may reflect a protective strategy that helps limit energy expenditure, reduce ROS accumulation, and safeguard the photosynthetic apparatus from damage (Chinnusamy and Zhu, 2009; Lawlor and Tezara, 2009).

### Enrichment of drought memory gene reveals genotype-specific pathway activation

Enrichment analysis of distinct drought memory genes identified in the leaf transcriptome of each genotype revealed genotype-specific pathway activation, highlighting divergent strategies of drought adaptation. This is consistent with findings in other studies that have demonstrated unique genetic strategies employed by differing varieties (Reynolds *et al*., 2005; Monneveux *et al*., 2012; Mwadzingeni *et al*., 2017; Bapela *et al*., 2022). We observed differences in the oxidative stress management, with ‘Intro’ displaying significant enrichment of pathways related to oxidoreductase activities, particularly those acting on NAD(P)H and aldehyde or oxo groups, as well as monooxygenase activity, suggesting a robust capacity for ROS detoxification (Reis *et al*., 2021; Das and Sen, 2024; Roth *et al*., 2024). Using a distinct gene expression profile, ‘Sonalika’ showed limited enrichment in oxidative stress-related pathways, with some indications of FAD and NADP binding. These features may offer some support for ROS management, but suggest a comparatively weaker antioxidant response. These findings agree with those of Pradhan and Chakraborty (2013) who, using the same genotype, reported decreased SOD and CAT activity in drought conditions, potentially undermining cellular integrity during drought stress.

Distinctively, the ‘Intro’ genotype showed clear enrichment of jasmonate-amino synthetase activity and responses to multiple abiotic stimuli including karrikin, indicating a sophisticated hormonal regulation during drought memory formation. In addition, other pathways such as response to osmotic stress, regulation of response to stimulus, ion transmembrane transporter activity, and small molecule biosynthetic process, commonly co-regulated with ABA or activated downstream of ABA signaling during water deficit were enriched. For example, osmotic stress responses often require ABA-induced transcriptional reprogramming (Zhang *et al*., 2006; Daszkowska-Golec, 2016; Muhammad Aslam *et al*., 2022). The concurrent enrichment of ion homeostasis and amino acid catabolism pathways in ‘Intro’ also reflects the metabolic reconfiguration role of ABA during dehydration stress (Urano *et al*., 2009; Roychoudhury and Basu, 2021). Jasmonic acid and related signaling molecules play pivotal roles in mediating stress responses and priming tolerance mechanisms (Raza *et al*., 2021; Sheteiwy et al., 2022). Notably, the presence of jasmonate-related activity suggests possible ABA/jasmonic acid hormonal crosstalk, a known component of long-term drought adaptation and memory (Chaves *et al*., 2002; Lawlor and Tezara, 2009). Meanwhile, ‘Intro’ plants accumulated more ABA in D1C2 seedlings, promoting stomatal closure (Supplementary Fig. S4A). In contrast, ‘Sonalika’ lacked enrichment in key phytohormone pathways, implying a less dynamic hormonal modulation that may reduce its capacity to fine-tune drought-responsive signaling networks (Salvi *et al*., 2021; El Sabagh *et al*., 2022). Concordantly, under drought, ABA content in ‘Sonalika’ plants with a history of drought was lower than in the control plants experiencing drought for the first time (Supplementary Fig. S4). This divergence in physiological strategy highlights multiple, possibly parallel, epigenetic and transcriptional routes to transgenerational tolerance (Lang-Mladek *et al*., 2010; Lämke and Bäurle, 2017).

Another most notable difference between the two genotypes was observed in the downstream translation and protein assembly machinery. ‘Intro’ exhibited strong enrichment of ribosomal components, translation, peptide biosynthesis pathways, and protein targeting and import mechanisms. This indicates a sustained capacity for efficient protein synthesis during drought, probably supporting the rapid production of stress-responsive proteins crucial for adaptation and stress memory (Fotovat *et al*., 2017). Such observations reinforce the hypothesis that an enhanced capacity for protein synthesis is a key drought adaptation strategy in wheat (Moloi *et al*., 2024). Unlike ‘Intro’, ‘Sonalika’ showed enrichment in pathways related to stress-induced proteostasis, including protein transport, protein localization, protein import, intracellular protein transmembrane transport, and macromolecule localization, with limited activation of ribosomal- and translation-related processes, which may compromise translational efficiency under stress. Instead, it employs a more reactive stress management strategy, where it mobilizes protective mechanisms to damaged or mislocalized proteins to mitigate damage and prepare for potential recurring stress, despite lacking inherent drought tolerance (Augustine, 2016; Su *et al*., 2019; Aghaie and Tafreshi, 2020).

Pathways involved in cation transport and ion homeostasis were notably enriched in ‘Intro’, underscoring the importance of maintaining ionic balance and osmotic regulation during drought (Cuin and Shabala, 2007; Shabala and Cuin, 2008). This capacity is crucial for preserving cellular turgor and enzyme function under water deficit (Li *et al*., 2021). Moreover, this genotype demonstrated enrichment of pathways related to cellular component assembly and lipid transport, including sterol and cholesterol metabolism, and lipid biosynthetic process, which are biochemical adaptations that are likely to support membrane integrity and fluidity, contributing to structural resilience under drought (Upchurch, 2008). Specifically, sterols and other structural lipids help stabilize lipid bilayers, reducing membrane phase transitions and leakage under water-limiting conditions. While ‘Sonalika’ showed some response to metal ions, it lacked specific enrichment for ion transport pathways, which may impair its ability to regulate cellular ion homeostasis during drought. In contrast, its enrichment leaned towards peroxisomal organization and immune-related responses, which are typically associated with oxidative stress detoxification and pathogen defense (Sandalio *et al*., 2013; Su *et al*., 2019). While this may reflect a responsive mechanism to cellular damage, it also indicates a shift toward reactive protection rather than proactive structural adaptation. As highlighted by Sandalio *et al*. (2013) and Smertenko (2017), peroxisomal remodeling under stress is often linked to elevated ROS processing and immune signaling, which are crucial but metabolically expensive.

Further, enrichment of pathways linked to carbohydrate biosynthesis and monosaccharide metabolism in ‘Intro’ suggests an active strategy for accumulating soluble sugars that support osmotic balance and energy homeostasis during drought (Gautam *et al*., 2022; J. Yu *et al*., 2025). Soluble sugars such as sucrose and glucose not only act as osmoprotectants, stabilizing proteins and membranes, but also serve as readily mobilizable energy reserves to fuel stress responses. According to Keunen *et al*. (2013), sugars also play a critical role in redox homeostasis, acting as signaling molecules that trigger antioxidant defenses. It also exhibits strong enrichment in proline metabolism, including proline metabolic process, proline catabolic process to glutamate, 1-pyrroline-5-carboxylate dehydrogenase activity, and glutamate metabolic process. The inclusion of catabolic terms, especially proline catabolism to glutamate, is critical, as it indicates controlled turnover of proline, supporting energy generation and cellular homeostasis, rather than just accumulation (Szabados and Savouré, 2010). However, ‘Sonalika’ portrayed a possible deficit in osmotic adjustment via sugar metabolism, and lacked any significant enrichment in proline metabolism, biosynthesis, or catabolic pathways, reflecting absence of metabolic reprogramming involving proline, and a general failure to engage amino acid-based stress mitigation strategies, especially those involving glutamate and proline cycling.

On the other hand, ‘Sonalika’ showed stronger evidence of transcriptional and post-transcriptional activity, with enriched pathways related to RNA processing and gene expression regulation, including catalytic activity acting on a nucleic acid, catalytic activity acting on RNA, tRNA metabolic process, non-coding RNA metabolic process, tRNA aminoacylation, seryl-tRNA aminoacylation, and serine-tRNA ligase activity. These pathways suggest that histone acetylation modifications terms identified in the seed transcriptome of ‘Sonalika’ set the stage for the broad transcriptional and RNA-level responses implied from the leaf transcriptome, potentially reflecting a reactive reprogramming of the transcriptome in response to drought. These findings agree with those of Shaar-Moshe *et al*. (2015), which highlight that genotypes with broad transcriptional responses often activate a wide range of drought-related processes. This broad activation enables a more dynamic and flexible stress response, enhancing the ability of the plant to cope with environmental fluctuations. However, the study also emphasizes that such wide-ranging transcriptional changes are not uniformly beneficial, as there is a trade-off between breadth of response and energy/resource allocation. The involvement of tRNA aminoacylation and RNA processing may indicate efforts to reboot the protein synthesis machinery under stress, but possibly with high energy cost and limited efficiency. Interestingly, ‘Intro’ shows limited direct enrichment in transcription-related terms in leaf tissue, supporting its seed-specific suggested epigenetic priming characterized by enrichment of histone methylation genes, and aligning with the concept of transcriptional priming, which allows plants to minimize large-scale transcriptional reprogramming (Crisp *et al*., 2016; Lämke and Bäurle, 2017). Therefore, while ‘Intro’ primarily exhibited pathways specifically tailored toward abiotic stress adaptation and drought-focused responses, ‘Sonalika’ presented a reactive, broader, and more generalized stress response profile. Surprisingly, both genotypes displayed evidence of similar physiological responses such as photochemical quantum yield of PSII, root surface area, and root crossings, despite their differing epigenetic landscapes (Supplementary Figs S1, S5, S6). Hence, even in the absence of enrichment of genes linked to heritable epigenetic marks, ‘Sonalika’ maintains adaptive potential by leveraging broad transcriptional networks that respond swiftly to environmental cues, ensuring survival and functional tolerance (Hilker *et al*., 2016; Lämke and Bäurle, 2017). Notably, it demonstrated increased stomatal conductance and assimilation rate in the D1C2D3 generation under drought (Supplementary Figs S2, S3), an indicator of improved gas exchange and photosynthetic efficiency (Wang *et al*., 2021).

### Conclusion and implications for drought memory and plant breeding

The results underscore a complex interplay among transcriptional, physiological, and potentially epigenetic adaptations, which manifest distinctly in the drought-tolerant ‘Intro’ and drought-sensitive ‘Sonalika’ wheat genotypes. ‘Intro’ displayed enrichment of transcripts linked to histone methylation, alongside stable transcriptional regulation, robust hormonal signaling, and coordinated biochemical adjustments that help maintain osmotic balance, oxidative stress tolerance, and structural integrity. In contrast, ‘Sonalika’ mounted a more transient but broad transcriptional response, marked by enrichment of transcripts associated with histone acetylation and pathways related to proteostasis and immune responses, suggesting an alternative stress management strategy that may not rely on methylation-related regulation. However, further investigation is required to elucidate the precise epigenetic and transcriptional mechanisms that enable these responses.

The exhibition of intergenerational and transgenerational drought memory in both genotypes, despite relying on a distinct molecular route, emphasizes that diverse regulatory pathways can converge to support long-term stress acclimation. These findings highlight not only the complexity of plant adaptive responses but also their practical relevance for breeding, which is reflected in genotype-specific transcriptional frameworks, potentially supported by histone-modifying enzymes, that shape strategies for energy management, stress endurance, and recovery. Notably, the presence of transgenerational stress memory in ‘Sonalika’, despite the absence of transcripts linked to classical methylation-based regulation, points to transcriptional plasticity and chromatin accessibility via acetylation as a viable compensatory mechanism, which could be leveraged in breeding programs.

Importantly, the drought memory genes identified in seeds and leaves provide actionable targets for molecular breeding strategies. These genes, together with associated physiological traits such as ABA accumulation, osmoprotectant levels, and antioxidant activity, could inform marker-assisted or genomic selection approaches aimed at developing wheat varieties with enhanced resilience to repeated drought stress. Selecting for either stable epigenetic priming or flexible transcriptional responsiveness, or combining both, offers a path toward cultivating varieties that balance stability and adaptability under variable environmental conditions.

However, this study is not without limitations. Although our data point to divergent transcriptional and physiological strategies, the focus on two genotypes and transcript-level inference of epigenetic regulation may limit broader generalization. Future research combining functional validation of candidate memory genes with direct measurements of histone modifications across a wider genetic pool and under field conditions will be essential to fully elucidate the mechanisms underlying stress memory and to translate these insights into practical breeding outcomes.

## Supplementary Material

erag203_Supplementary_Data

## Data Availability

All related data can be found in the manuscript and its supplementary data. Sequencing facility identifiers differ from manuscript sample names. A full correspondence between manuscript sample IDs and sequencing identifiers is provided in Supplementary Table S1. All MACE RNA-seq data generated in this study have been deposited in the European Nucleotide Archive (ENA) under project accession number PRJEB108653. Associated metadata are available within the ENA submission. Further inquiries can be directed to the corresponding author.

## Supplementary data

The following supplementary data are available at *JXB* online.

Table S1. Library information including total sequences and mapping statistics.

Table S2. Functional annotation of top-ranked inter- and transgenerational memory genes identified in ‘Intro’ and ‘Sonalika’.

Fig. S1. Photochemical quantum yield of PSII in wheat under drought and control conditions across seedlings from different seed set categories.

Fig. S2. Stomatal conductance in ‘Intro’ and ‘Sonalika’ wheat genotypes under drought and control conditions across seedlings from different seed set categories.

Fig. S3. Assimilation rate in ‘Intro’ and ‘Sonalika’ wheat genotypes under drought and control conditions across seedlings from different seed set categories

Fig. S4. Abscisic acid content in ‘Intro’ and ‘Sonalika’ wheat genotypes under drought and control conditions across seedlings from different seed set categories.

Fig. S5. Root surface area in ‘Intro’ and ‘Sonalika’ wheat genotypes under drought and control conditions across seedlings from different seed set categories.

Fig. S6. Root crossings in ‘Intro’ and ‘Sonalika’ wheat genotypes under drought and control conditions across seedlings from different seed set categories.

Fig. S7. Correlation-based clustering of distinct intergenerational drought memory genes in ‘Intro’ annotated by response pattern.

Fig. S8. Correlation-based clustering of distinct transgenerational drought memory genes in ‘Intro’ annotated by response pattern.

Fig. S9. Correlation-based clustering of distinct intergenerational drought memory genes in ‘Sonalika’ annotated by response pattern.

Fig. S10. Correlation-based clustering of distinct transgenerational drought memory genes in ‘Sonalika’ annotated by response pattern.

Dataset S1. Four numbered worksheets, each beginning with a brief description of its contents, including genotype, memory type, and an explanation of the trait correlation data.
