# Supplementary material for: Comparative transcriptomics reveals epigenetic and transcriptional regulatory genes associated with drought stress memory in winter wheat
Source: J Exp Bot. 2026 Apr 29;77(16):5284–304. doi: 10.1093/jxb/erag203 (PMC13529338; doi:10.1093/jxb/erag203)
Supplement: Supplementary file 1 [file erag203_supplementary_data.zip › JEXBOT316030-file001.pdf]

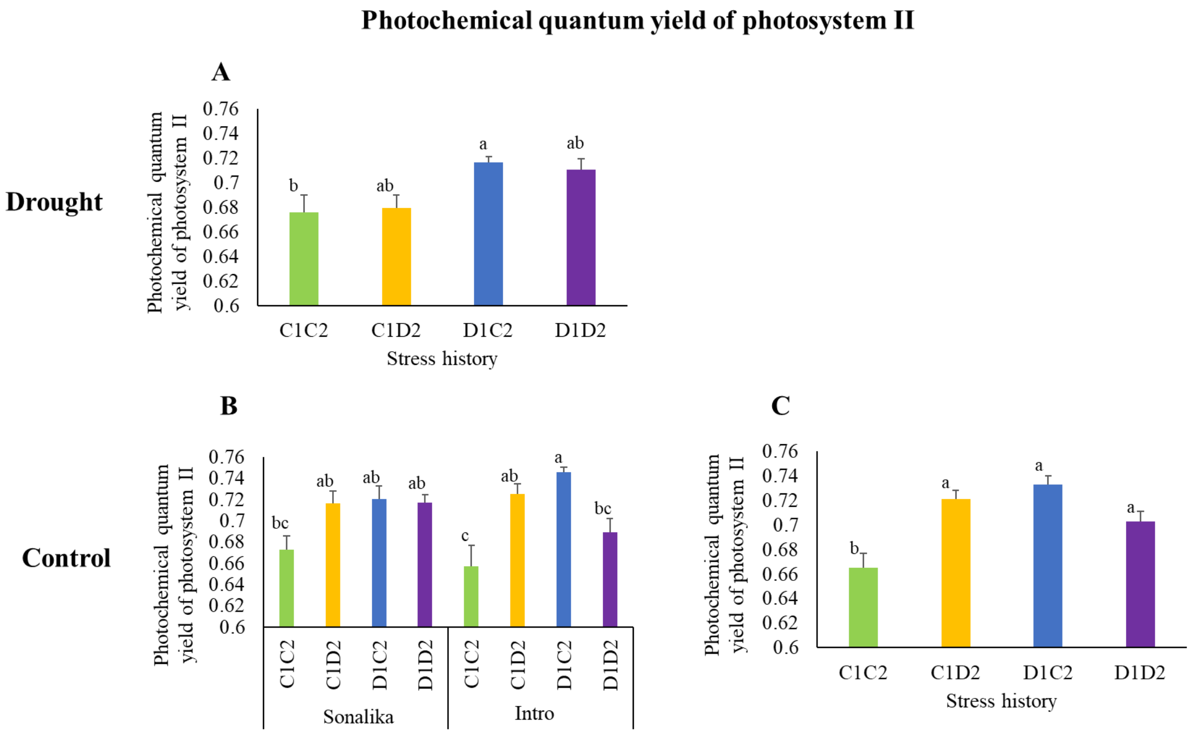

Fig. S1: Photochemical quantum yield of photosystem II in wheat under drought and under control. The statistical comparisons are made for control treatments between the trait mean values across the different seed set categories (C1C2, C1D2, D1C2 and D1D2) for each genotype separately (**B**) and combined (A and C) under both conditions. The letters indicate which categories are significantly different from one another based on the Tukey test ($p < 0.05$). Categories with different letters are significantly different. Error bars represent the standard error of the mean.

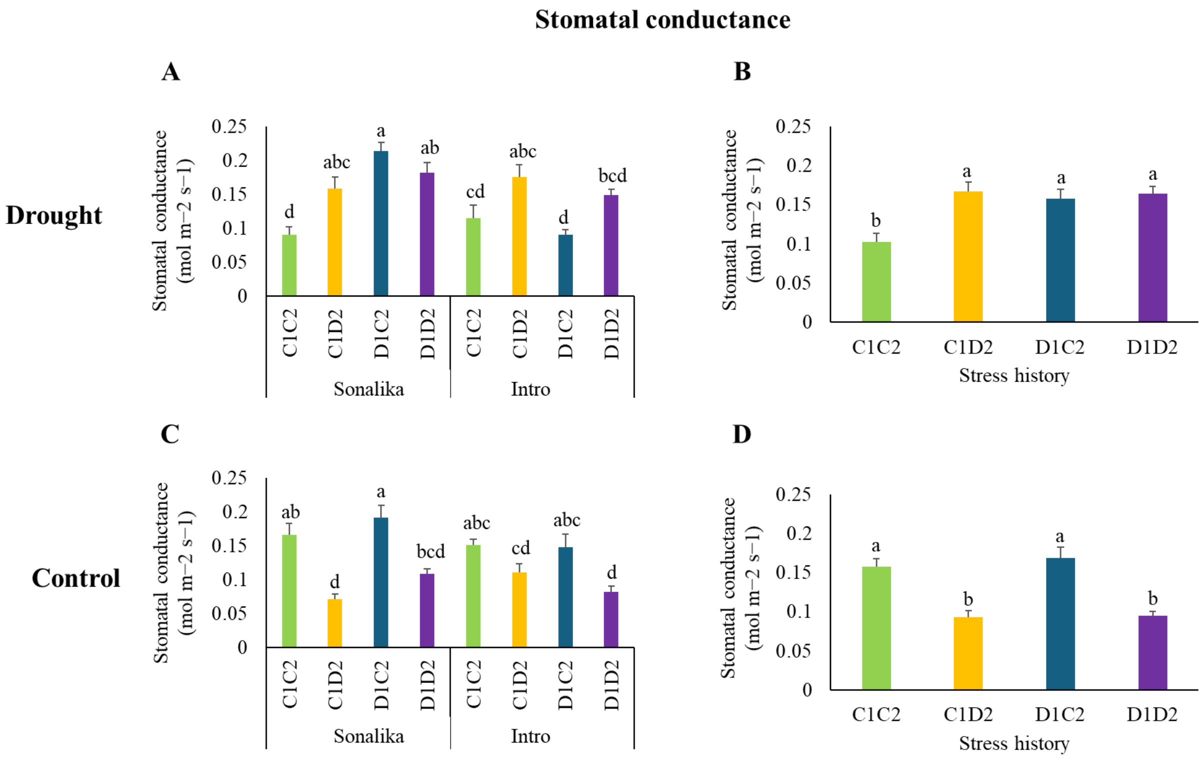

Fig. S2: Stomatal conductance measurements in "Intro" and "Sonalika" wheat genotypes. The statistical comparisons are made for both drought and control treatments between the trait mean values across the different seed set categories (C1C2, C1D2, D1C2 and D1D2) for each genotype separately (**A and C**) and combined (**B and D**). The letters indicate which categories are significantly different from one another based on the Tukey test ($p < 0.05$). Categories with different letters are significantly different. Error bars represent the standard error of the mean.

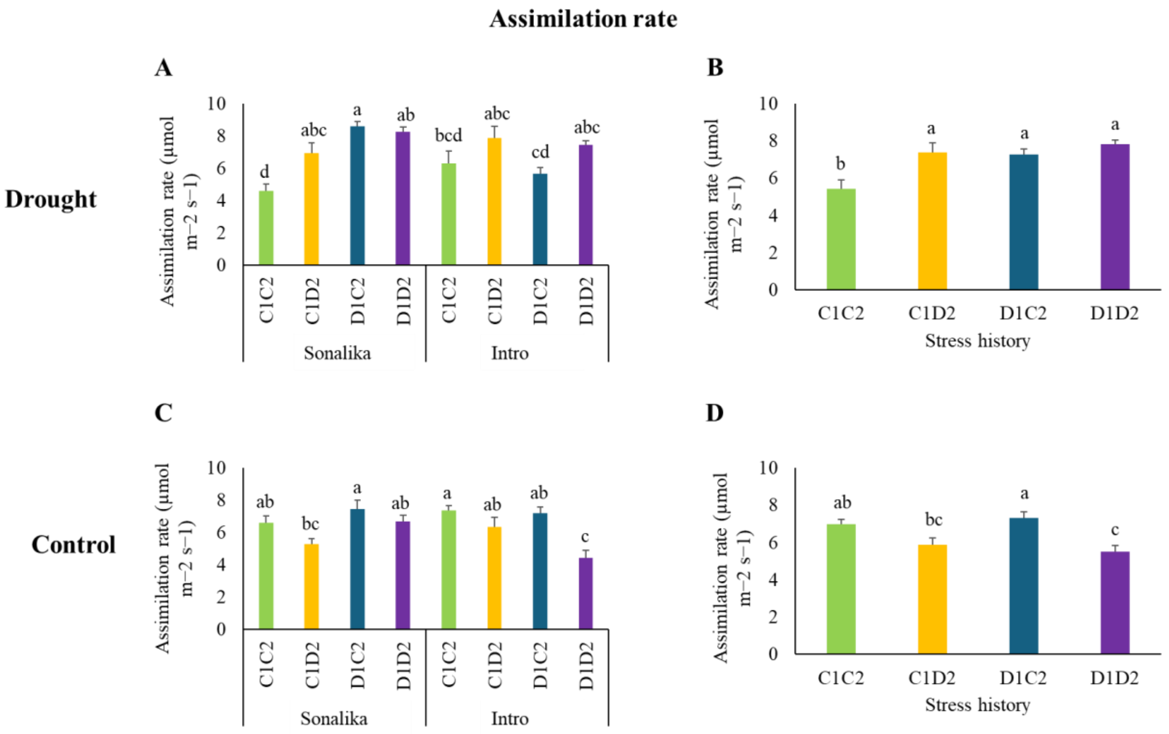

Fig. S3: Assimilation rate measurements in "Intro" and "Sonalika" wheat genotypes. The statistical comparisons are made for both drought and control treatments between the trait mean values across the different seed set categories (C1C2, C1D2, D1C2 and D1D2) for each genotype separately (**A and C**) and combined (**B and D**). The letters indicate which categories are significantly different from one another based on the Tukey test ($p < 0.05$). Categories with different letters are significantly different. Error bars represent the standard error of the mean.

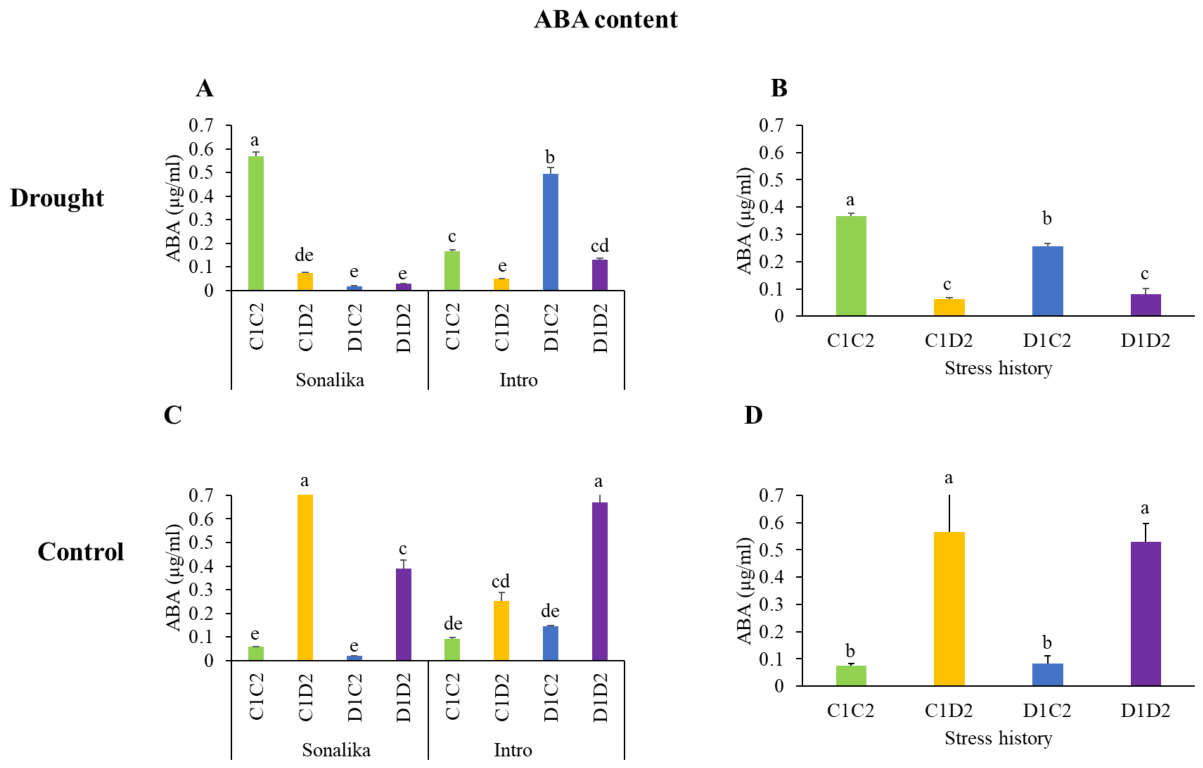

Fig. S4: ABA measurements in “Intro” and “Sonalika” wheat genotypes. The statistical comparisons are made for both drought and control treatments between the trait mean values across the different seed set categories (C1C2, C1D2, D1C2 and D1D2) for each genotype separately (**A and C**) and combined (**B and D**). The letters indicate which categories are significantly different from one another based on the Tukey test ($p < 0.05$). Categories that share the same letter do not have statistically significant differences in their mean values. Conversely, categories with different letters are significantly different.

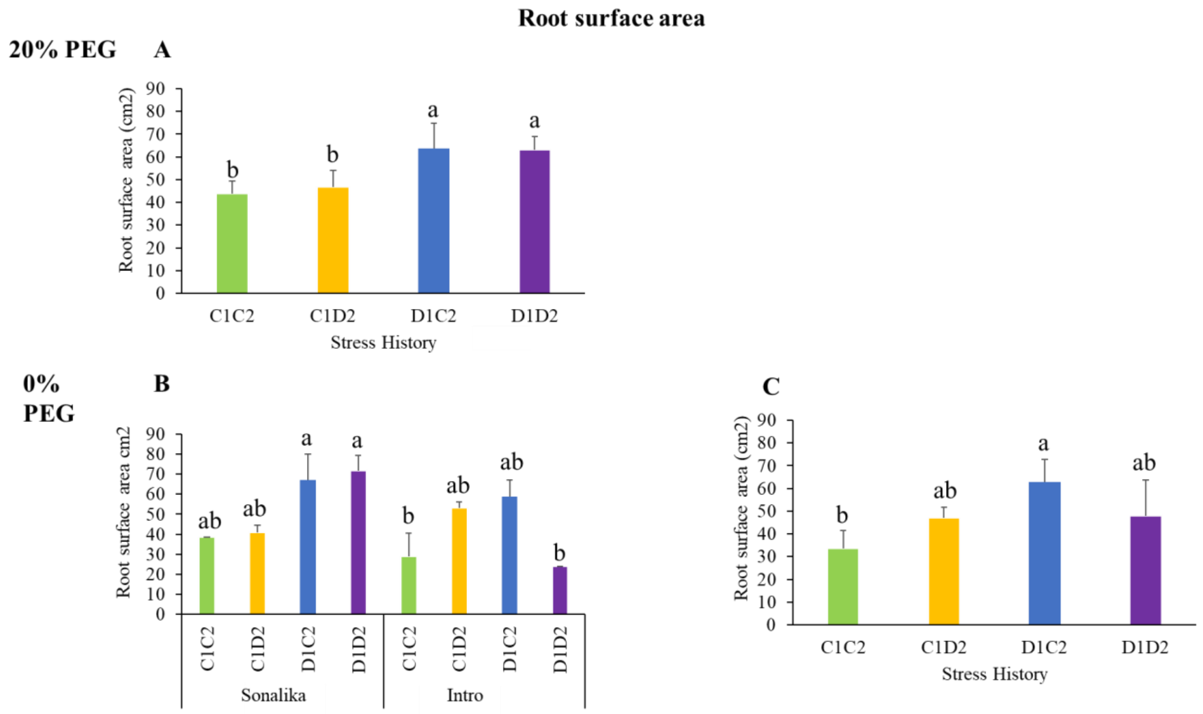

Fig. S5: Root surface area measurements in "Intro" and "Sonalika" wheat genotypes. The statistical comparisons are made for control treatments between the trait mean values across the different seed set categories (C1C2, C1D2, D1C2 and D1D2) for each genotype separately (B) and combined (A and C) under both conditions. The letters indicate which categories are significantly different from one another based on the Tukey test ($p < 0.05$). Categories with different letters are significantly different. Error bars represent the standard error of the mean.

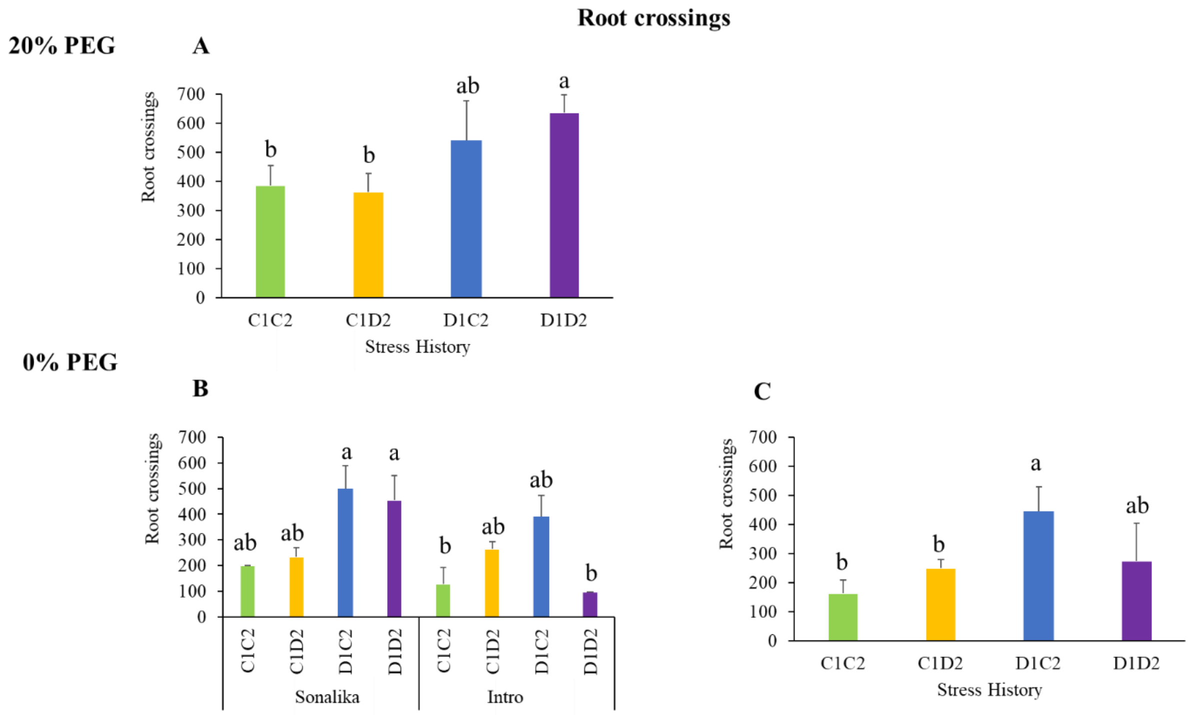

Fig. S6: Root crossings measurements in "Intro" and "Sonalika" wheat genotypes. The statistical comparisons are made for control treatments between the trait mean values across the different seed set categories (C1C2, C1D2, D1C2 and D1D2) for each genotype separately (B) and combined (A and C) under both conditions. The letters indicate which categories are significantly different from one another based on the Tukey test ($p < 0.05$). Categories with different letters are significantly different. Error bars represent the standard error of the mean.

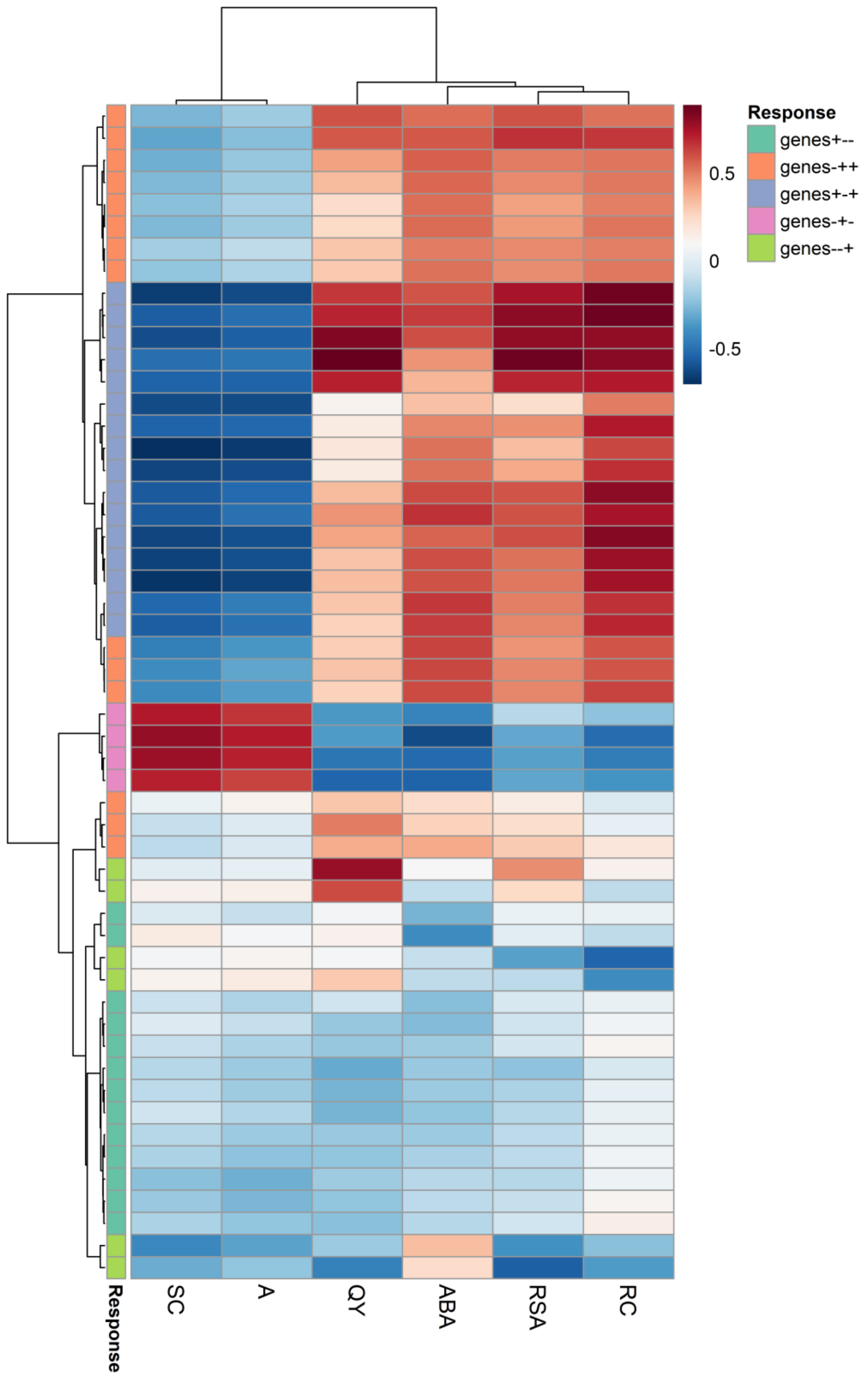

Fig. S7: Correlation-based clustering of distinct intergenerational drought memory genes in "Intro" annotated by response pattern. Each row represents a memory gene and each column a trait. Genes with similar correlation profiles cluster together, highlighting coordinated stress responses. Positive correlations are shown in red, negative correlations in blue. Full numeric data are provided in File S1

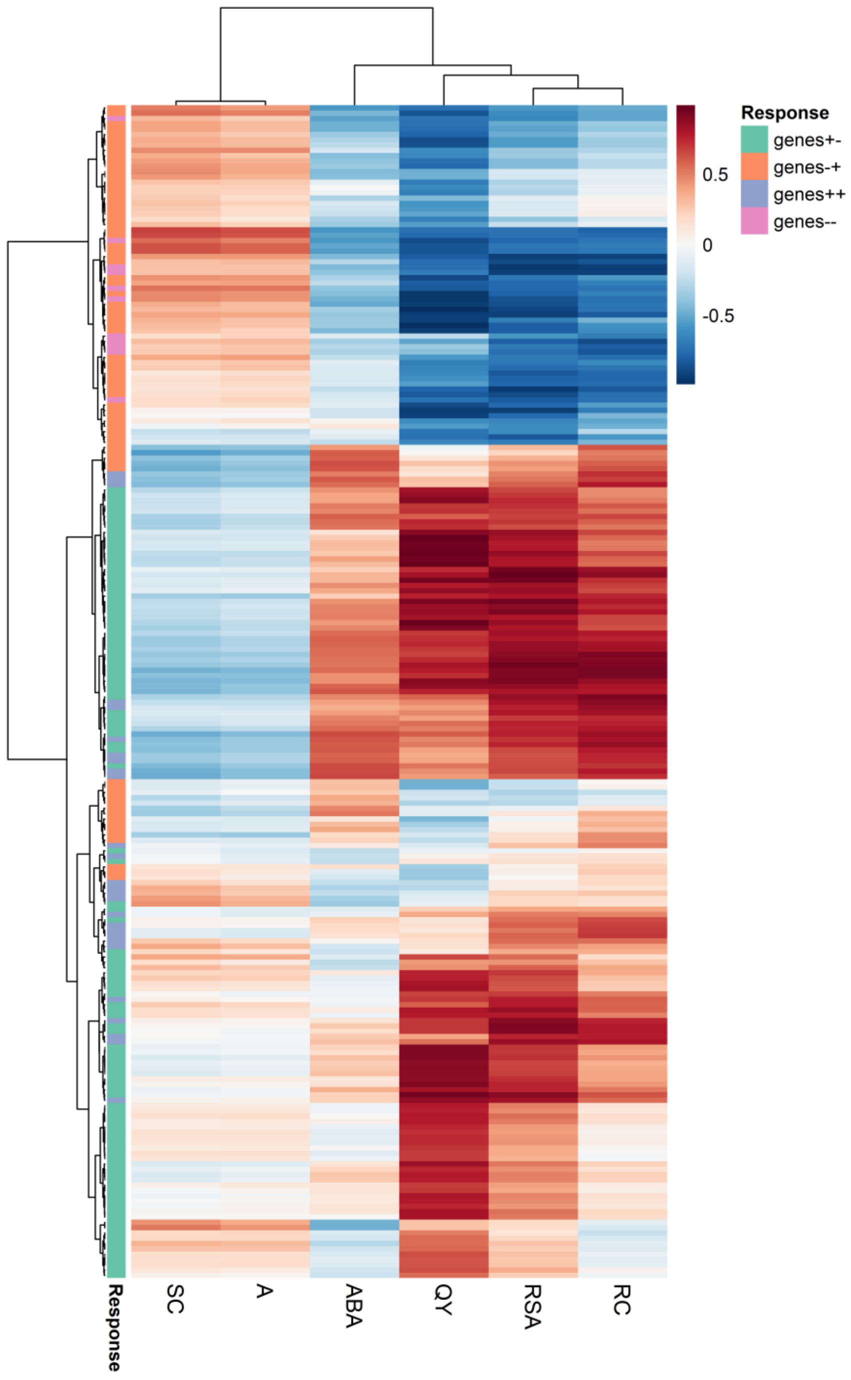

Fig. S8: Correlation-based clustering of distinct transgenerational drought memory genes in "Intro" annotated by response pattern. Each row represents a memory gene and each column a trait. Genes with similar correlation profiles cluster together, highlighting coordinated stress responses. Positive correlations are shown in red, negative correlations in blue. Full numeric data are provided in File S1

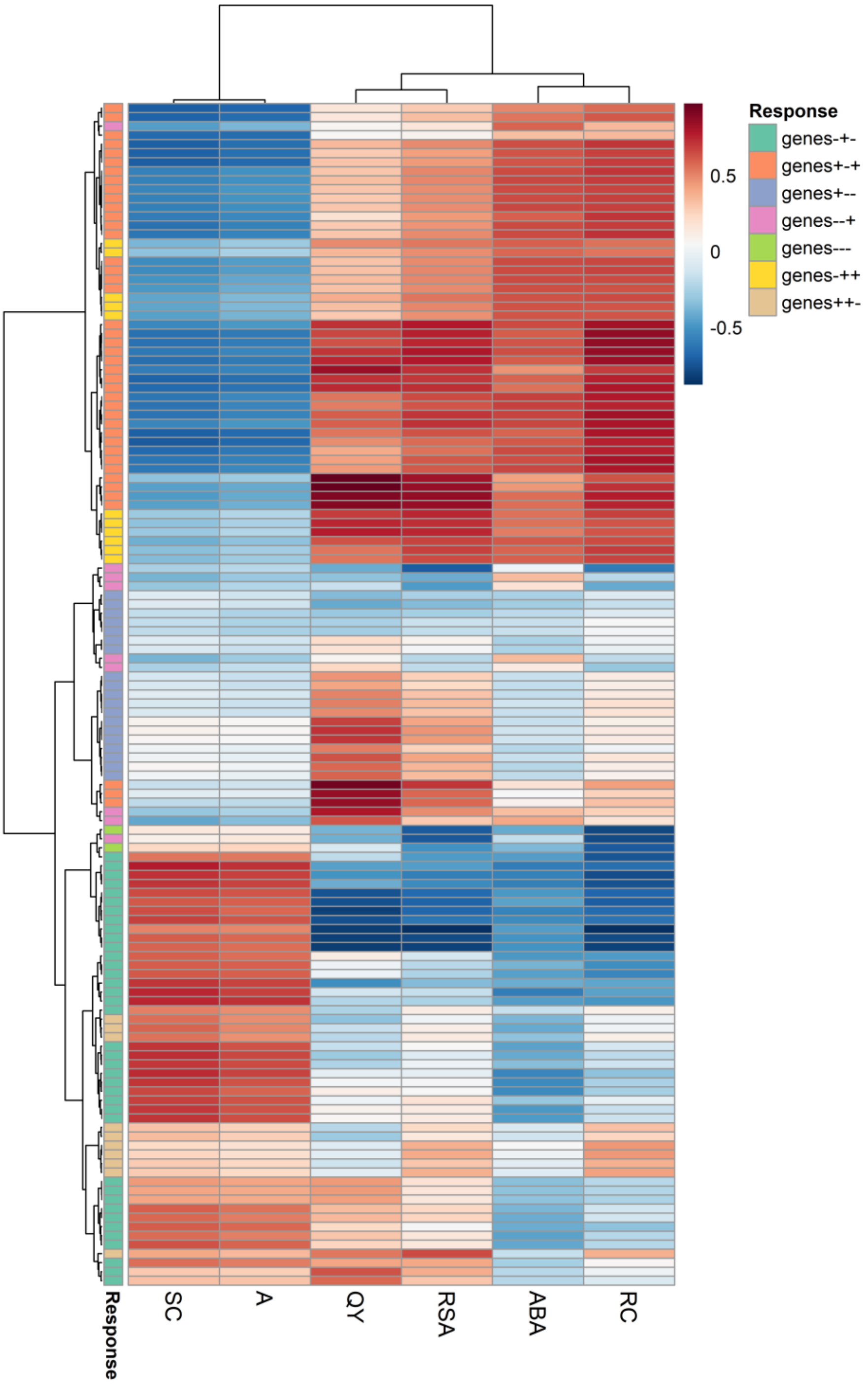

Fig. S9: Correlation-based clustering of distinct intergenerational drought memory genes in "Sonalika," annotated by response pattern. Each row represents a memory gene and each column a trait. Genes with similar correlation profiles cluster together, highlighting coordinated stress responses. Positive correlations are shown in red, negative correlations in blue. Full numeric data are provided in File S1

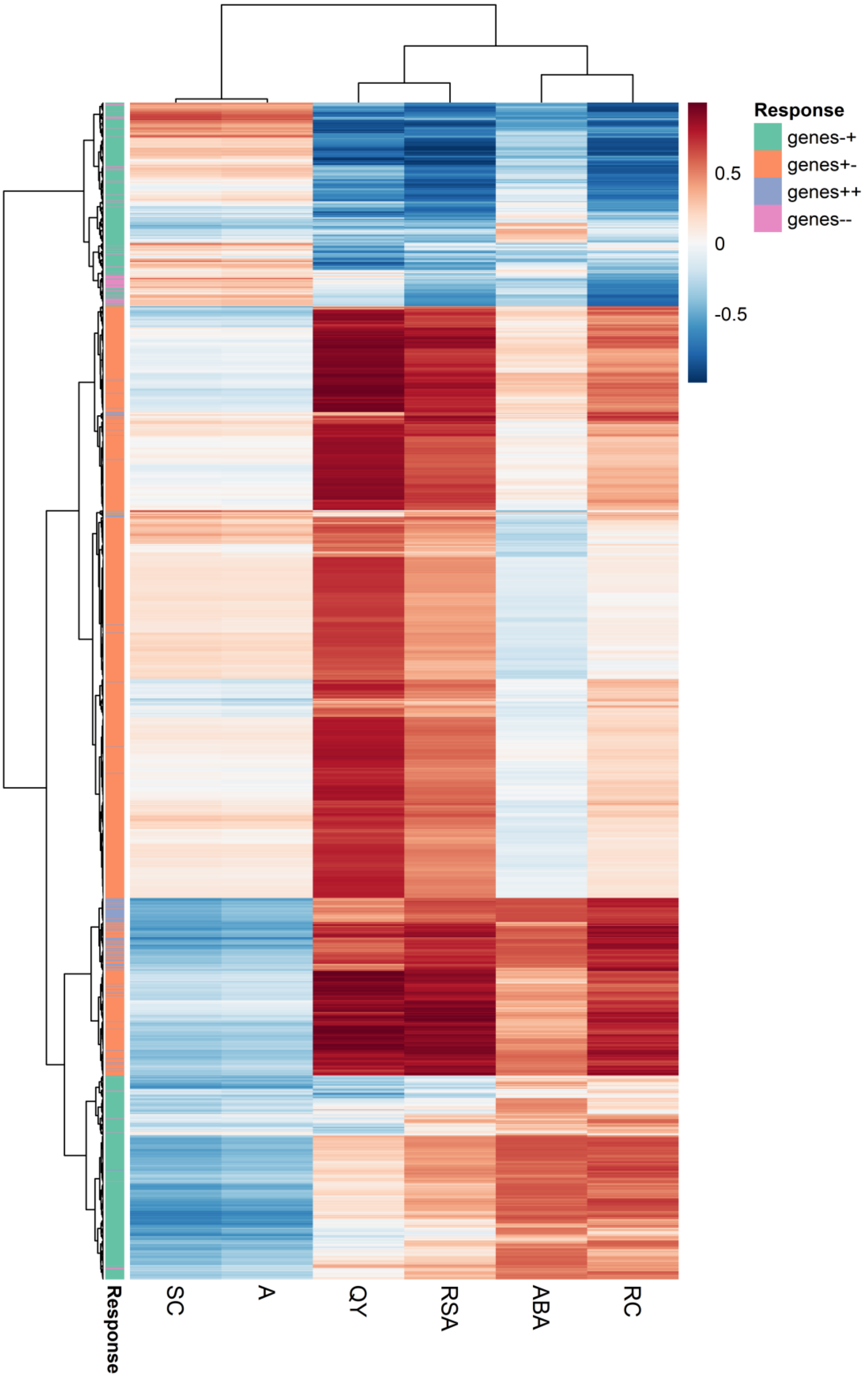

Fig. S10: Correlation-based clustering of distinct transgenerational drought memory genes in "Sonalika," annotated by response pattern. Each row represents a memory gene and each column a trait. Genes with similar correlation profiles cluster together, highlighting coordinated stress responses. Positive correlations are shown in red, negative correlations in blue. Full numeric data are provided in File S1

Table S1: Library information including total sequences and mapping statistics. For each sample, the table provides the genotype identifier (Genotype ID) and name (Genotype Name), tissue type, and biological replicate number. Sequencing depth is reported as total number of raw reads (Total Sequences) and read length (Sequence Length). The number of reads retained after quality filtering (Reads after filtering) and the number of reads successfully aligned to the reference genome (Mapped reads) are shown, together with the corresponding mapping rate (percentage of mapped reads relative to filtered reads). The reference genome used for alignment is indicated, and the ENA project accession number is provided for data availability and public access.

Genotype ID	Genotype Name	Tissue	Biological replicate	Total Sequences	Sequence Length	Reads after filtering	Mapped reads	Mapping rate	Reference genome	ENA Project accession number
**ID14**	Sonalika	Seed	rep1	5044424	76	4061375	2364490	47	IWGSC RefSeq v2.3	PRJEB108653
**ID14**	Sonalika	Seed	rep2	5546450	76	4496111	2730575	49	IWGSC RefSeq v2.3	PRJEB108653
**ID14**	Sonalika	Seed	rep3	5738206	76	4777289	2952140	51	IWGSC RefSeq v2.3	PRJEB108653
**ID5**	Intro	Seed	rep1	3589740	76	3037721	1800051	50	IWGSC RefSeq v2.3	PRJEB108653
**ID5**	Intro	Seed	rep2	4982303	76	4157351	2412834	48	IWGSC RefSeq v2.3	PRJEB108653
**ID14**	Sonalika	Seed	rep1	18110301	76	9969729	5182067	29	IWGSC RefSeq v2.3	PRJEB108653
**ID14**	Sonalika	Seed	rep2	6439813	76	4439763	2619251	41	IWGSC RefSeq v2.3	PRJEB108653
**ID14**	Sonalika	Seed	rep3	6590073	76	4718369	2722134	41	IWGSC RefSeq v2.3	PRJEB108653
**ID5**	Intro	Seed	rep1	6559175	76	5149462	3174654	48	IWGSC RefSeq v2.3	PRJEB108653
**ID5**	Intro	Seed	rep2	18685072	76	12597107	7654981	41	IWGSC RefSeq v2.3	PRJEB108653
**ID5**	Intro	Seed	rep3	6739320	76	5224619	3350254	50	IWGSC RefSeq v2.3	PRJEB108653
**ID14**	Sonalika	Leaf	rep1	5719826	76	3959154	2314582	40	IWGSC RefSeq v2.3	PRJEB108653
**ID14**	Sonalika	Leaf	rep2	6125412	76	3992019	1591161	26	IWGSC RefSeq v2.3	PRJEB108653
**ID14**	Sonalika	Leaf	rep3	1735562	76	1022343	557724	32	IWGSC RefSeq v2.3	PRJEB108653
**ID5**	Intro	Leaf	rep1	5469282	76	4879185	2807367	51	IWGSC RefSeq v2.3	PRJEB108653
**ID5**	Intro	Leaf	rep2	370542	76	231474	134365	36	IWGSC RefSeq v2.3	PRJEB108653
**ID5**	Intro	Leaf	rep3	10802749	76	9813053	5127255	47	IWGSC RefSeq v2.3	PRJEB108653
**ID14**	Sonalika	Leaf	rep1	11524486	76	10511165	5928973	51	IWGSC RefSeq v2.3	PRJEB108653
**ID14**	Sonalika	Leaf	rep2	10929968	76	7439395	3895167	36	IWGSC RefSeq v2.3	PRJEB108653
**ID14**	Sonalika	Leaf	rep3	5663512	76	4801183	3018837	53	IWGSC RefSeq v2.3	PRJEB108653
**ID5**	Intro	Leaf	rep1	12061551	76	11397991	5273496	44	IWGSC RefSeq v2.3	PRJEB108653

**ID5**	Intro	Leaf	rep2	5226106	76	4593015	2601875	50	IWGSC RefSeq v2.3	PRJEB108653
**ID5**	Intro	Leaf	rep3	7202358	76	6735312	3240860	45	IWGSC RefSeq v2.3	PRJEB108653
**ID14**	Sonalika	Leaf	rep1	6184925	76	4295566	2698710	44	IWGSC RefSeq v2.3	PRJEB108653
**ID14**	Sonalika	Leaf	rep2	9030492	76	6661136	4393736	49	IWGSC RefSeq v2.3	PRJEB108653
**ID14**	Sonalika	Leaf	rep3	10074908	76	8985804	5750394	57	IWGSC RefSeq v2.3	PRJEB108653
**ID5**	Intro	Leaf	rep1	13133913	76	7363959	3472876	26	IWGSC RefSeq v2.3	PRJEB108653
**ID5**	Intro	Leaf	rep2	10340917	76	9095921	4984508	48	IWGSC RefSeq v2.3	PRJEB108653
**ID5**	Intro	Leaf	rep3	11957610	76	7870442	3462748	29	IWGSC RefSeq v2.3	PRJEB108653
**ID14**	Sonalika	Leaf	rep1	11216013	76	9327451	5459879	49	IWGSC RefSeq v2.3	PRJEB108653
**ID14**	Sonalika	Leaf	rep2	7362145	76	6146253	3009076	41	IWGSC RefSeq v2.3	PRJEB108653
**ID14**	Sonalika	Leaf	rep3	11001826	76	9768942	4891743	44	IWGSC RefSeq v2.3	PRJEB108653
**ID5**	Intro	Leaf	rep1	13404493	76	12267869	7077451	53	IWGSC RefSeq v2.3	PRJEB108653
**ID5**	Intro	Leaf	rep2	14369077	76	13284108	7724317	54	IWGSC RefSeq v2.3	PRJEB108653
**ID5**	Intro	Leaf	rep3	12992496	76	12149456	6198164	48	IWGSC RefSeq v2.3	PRJEB108653
**ID14**	Sonalika	Leaf	rep1	7482424	76	5996986	3830140	51	IWGSC RefSeq v2.3	PRJEB108653
**ID14**	Sonalika	Leaf	rep2	6883963	76	5236718	3086311	45	IWGSC RefSeq v2.3	PRJEB108653
**ID14**	Sonalika	Leaf	rep3	3036653	76	1691776	882087	29	IWGSC RefSeq v2.3	PRJEB108653
**ID5**	Intro	Leaf	rep1	5458320	76	4702125	2476119	45	IWGSC RefSeq v2.3	PRJEB108653
**ID5**	Intro	Leaf	rep2	9126675	76	8168646	4054686	44	IWGSC RefSeq v2.3	PRJEB108653
**ID5**	Intro	Leaf	rep3	4583843	76	2554424	1415591	31	IWGSC RefSeq v2.3	PRJEB108653
**ID14**	Sonalika	Leaf	rep1	4970807	76	3846298	2318855	47	IWGSC RefSeq v2.3	PRJEB108653
**ID14**	Sonalika	Leaf	rep2	8001791	76	5296332	3027634	38	IWGSC RefSeq v2.3	PRJEB108653
**ID14**	Sonalika	Leaf	rep3	3334372	76	1530814	743133	22	IWGSC RefSeq v2.3	PRJEB108653
**ID5**	Intro	Leaf	rep1	6771907	76	5289367	2854472	42	IWGSC RefSeq v2.3	PRJEB108653
**ID5**	Intro	Leaf	rep2	3571290	76	1872751	1044126	29	IWGSC RefSeq v2.3	PRJEB108653
**ID5**	Intro	Leaf	rep3	3909938	76	1955852	1072115	27	IWGSC RefSeq v2.3	PRJEB108653
**ID14**	Sonalika	Leaf	rep1	9556460	76	6414625	3012645	32	IWGSC RefSeq v2.3	PRJEB108653
**ID14**	Sonalika	Leaf	rep2	14849603	76	7836754	3298906	22	IWGSC RefSeq v2.3	PRJEB108653
**ID14**	Sonalika	Leaf	rep3	28103781	76	22779366	2903783	10	IWGSC RefSeq v2.3	PRJEB108653

**ID5**	Intro	Leaf	rep1	14580789	76	12951844	6401657	44	IWGSC RefSeq v2.3	PRJEB108653
**ID5**	Intro	Leaf	rep2	7423897	76	6686499	3768185	51	IWGSC RefSeq v2.3	PRJEB108653
**ID5**	Intro	Leaf	rep3	7400349	76	6719242	3393283	46	IWGSC RefSeq v2.3	PRJEB108653
**ID14**	Sonalika	Leaf	rep1	17327681	76	8232075	3455923	20	IWGSC RefSeq v2.3	PRJEB108653
**ID14**	Sonalika	Leaf	rep2	7792929	76	5397882	2530617	32	IWGSC RefSeq v2.3	PRJEB108653
**ID14**	Sonalika	Leaf	rep3	5662707	76	2332490	448036	8	IWGSC RefSeq v2.3	PRJEB108653
**ID5**	Intro	Leaf	rep1	9418658	76	7747350	4098205	44	IWGSC RefSeq v2.3	PRJEB108653
**ID5**	Intro	Leaf	rep2	9565114	76	8693980	4404069	46	IWGSC RefSeq v2.3	PRJEB108653
**ID5**	Intro	Leaf	rep3	2699469	76	1686621	960795	36	IWGSC RefSeq v2.3	PRJEB108653
**ID5**	Intro	Seed	rep3	12781632	76	9393190	5713154	45	IWGSC RefSeq v2.3	PRJEB108653
**ID14**	Sonalika	Seed	rep1	6240720	76	5014413	2969164	48	IWGSC RefSeq v2.3	PRJEB108653
**ID14**	Sonalika	Seed	rep2	6520827	76	5125766	2844618	44	IWGSC RefSeq v2.3	PRJEB108653
**ID14**	Sonalika	Seed	rep3	6810189	76	5413550	3265232	48	IWGSC RefSeq v2.3	PRJEB108653
**ID5**	Intro	Seed	rep1	7276197	76	5425106	3510310	48	IWGSC RefSeq v2.3	PRJEB108653
**ID5**	Intro	Seed	rep2	9141385	76	6986882	4293758	47	IWGSC RefSeq v2.3	PRJEB108653
**ID5**	Intro	Seed	rep3	6447521	76	5024064	3080771	48	IWGSC RefSeq v2.3	PRJEB108653
**ID14**	Sonalika	Seed	rep1	6976489	76	5284044	3195487	46	IWGSC RefSeq v2.3	PRJEB108653
**ID14**	Sonalika	Seed	rep2	5480989	76	4452860	2762672	50	IWGSC RefSeq v2.3	PRJEB108653
**ID14**	Sonalika	Seed	rep3	14010691	76	9553590	5635872	40	IWGSC RefSeq v2.3	PRJEB108653
**ID5**	Intro	Seed	rep1	6818010	76	5175370	3137774	46	IWGSC RefSeq v2.3	PRJEB108653
**ID5**	Intro	Seed	rep2	5100931	76	4220365	2467993	48	IWGSC RefSeq v2.3	PRJEB108653
**ID5**	Intro	Seed	rep3	6471922	76	5064572	2989452	46	IWGSC RefSeq v2.3	PRJEB108653

Table S2: Functional annotation of top ranked distinct inter and transgenerational memory genes identified in "Intro" and "Sonalika". The table lists each gene identifier (Gene ID), any alternative names, putative function, and, where available, orthologs in *Arabidopsis thaliana*. Genes were selected based on their mean absolute correlation with six morpho-physiological traits (quantum yield, stomatal conductance, assimilation rate, ABA content, root surface area, root crossings).

**Gene ID**	**Alternative name(s) if any**	**Function**	**Ortholog name (Arabidopsis thaliana) if available**
**Intro intergenerational memory genes top correlated with the traits**			
TRAESCS3D02G509100		encodes NAD(P)H-quinone oxidoreductase subunit O, chloroplastic	NdhO (AT1G74880)
TRAESCS1D02G109800	MUB1	encodes Ubiquitin-40S ribosomal protein S27a	UBQ6 (AT2G47110)
TRAESCS1B02G296600		encodes uncharacterized protein LOC109746576	
TRAESCS2A02G335200		encodes Indole-3-glycerol phosphate synthase, chloroplastic	AT2G04400 (AT2G04400)
TRAESCS5B02G519500		encodes uncharacterized protein LOC123114198	AT4G09620 (AT4G09620)
TRAESCS2B02G070800		encodes hypothetical protein CFC21_057613	
TRAESCS5D02G115300	RPS29	encodes 40S ribosomal protein S29	AT3G43980 (AT3G43980)
TRAESCS3B02G179500	CYP71A1	encodes Cytochrome P450 71A2	
TRAESCS1D02G216300		encodes uncharacterized protein LOC123181631	AT2G44870 (AT2G44870)
TRAESCS4A02G435600		encodes uncharacterized protein LOC123088279	AT4G35080 (AT4G35080)
TRAESCS1A02G367500		encodes uncharacterized protein LOC123065781	
TRAESCS3A02G432700		encodes hypothetical protein CFC21_037634	

TRAESCS4B02G341400	CBP1, SCPL1, SCPL10, SCPL11, SCPL13, SCPL14, SCPL15, SCPL16, SCPL17, SCPL18, SCPL19, SCPL2, SCPL3, SCPL4, SCPL5, SCPL6, SCPL7, SCPL9, SNG1	encodes Serine carboxypeptidase-like 18	scpl18 (AT1G33540)
TRAESCS3D02G048200		encodes hypothetical protein CFC21_045595	
TRAESCS1D02G149700		encodes Ribonuclease 1	
TRAESCS3D02G038000	MTP7	encodes Metal tolerance protein 7	AT1G79520 (AT1G79520)
TRAESCS1A02G357900	FIS1	encodes Probable aldehyde dehydrogenase	ALDH12A1 (AT5G62530)
TRAESCS2A02G467200		encodes uncharacterized protein LOC123186126	
TRAESCS3B02G172400	GH3.12	encodes Jasmonoyl--L-amino acid synthetase JAR4	JAR1 (AT2G46370)
TRAESCS5A02G391500		encodes Senescence-specific cysteine protease SAG39	SAG12 (AT5G45890)
**Intro transgenerational memory genes top correlated with the traits**			
TRAESCS3D02G196800		encodes RAB11-binding protein RELCH homolog	AT5G16210 (AT5G16210)
TRAESCS3D02G394300		encodes RAN GTPase-activating protein 1	
TRAESCS4D02G133100		encodes Caffeoylshikimate esterase	AT2G39410 (AT2G39410), AT2G39420 (AT2G39420), AT3G55180 (AT3G55180), AT3G55190 (AT3G55190)
TRAESCS4B02G297500		encodes 3-ketoacyl-CoA synthase 10	KCS10 (AT2G26250)
TRAESCS4B02G144200	TCTP	encodes Translationally controlled tumor protein homolog	AT3G05540 (AT3G05540), TCTP (AT3G16640)
TRAESCS4B02G161300	IMP3	encodes Inositol monophosphatase 3	VTC4 (AT3G02870)
TRAESCS5A02G071800	CYP81E9	encodes Cytochrome P450 81Q32	

TRAESCS3A02G415300		encodes uncharacterized protein LOC119270010	AT1G52910 (AT1G52910), AT1G61065 (AT1G61065), AT3G15480 (AT3G15480), AT4G27435 (AT4G27435)
TRAESCS5A02G124700	CBL6	encodes Calcineurin B-like protein 6	
TRAESCS2B02G233300	THF1	encodes Protein THYLAKOID FORMATION1	
TRAESCS3D02G144400		encodes ABC transporter D family member 2, chloroplastic	ABCD2 (AT1G54350)
TRAESCS4D02G339700		encodes 50S ribosomal protein L5, chloroplastic	AT4G01310 (AT4G01310)
TRAESCS3D02G340700	DEE76	encodes Putative MO25-like protein At5g47540	AT5G18940 (AT5G18940)
TRAESCS4B02G109900	ALDP, FBA1	encodes Fructose-bisphosphate aldolase, chloroplastic	FBA1 (AT2G21330), FBA2 (AT4G38970)
TRAESCS3D02G240200		encodes CASP-like protein 4A3	
TRAESCS1A02G411900		encodes Ketol-acid reductoisomerase, chloroplastic	AT3G58610 (AT3G58610)
TRAESCS3A02G383100	XRN2	encodes 5'-3' exoribonuclease 3	XRN3 (AT1G75660)
TRAESCS2A02G272100		encodes Oligoribonuclease	AT2G26970 (AT2G26970)
TRAESCS4D02G329400	HVA1	encodes Late embryogenesis abundant protein 19	
TRAESCS1A02G265200	SWEET1B	encodes Bidirectional sugar transporter SWEET1b	SWEET1 (AT1G21460)
**Sonalika intergenerational memory genes top correlated with the traits**			
TRAESCS3B02G137900	Embryo defective 2410	encodes Protein SUBSTANDARD STARCH GRAIN 4, chloroplastic	emb2410 (AT2G25660)
TRAESCS2B02G464400		encodes Late embryogenesis abundant protein At5g17165	
TRAESCS4B02G032200		encodes uncharacterized protein LOC123090736	AT1G55265 (AT1G55265)
TRAESCS5B02G209700		encodes Putative gamma-glutamylcyclotransferase At3g02910	AT5G46720 (AT5G46720)

TRAESCS1A02G200900		encodes Probable helicase MAGATAMA 3	AT1G65780 (AT1G65780), AT1G65810 (AT1G65810), AT4G05540 (AT4G05540), AT5G37030 (AT5G37030)
TRAESCS4D02G191500		encodes Uncharacterized protein At5g01610	AT3G08890 (AT3G08890), AT5G37070 (AT5G37070)
TRAESCS2B02G554600		encodes Protein MIZU-KUSSEI 1	AT4G39610 (AT4G39610)
TRAESCS2B02G154300		encodes hypothetical protein CFC21_022072	
TRAESCS2A02G111100		encodes uncharacterized protein LOC119358981	
TRAESCS3A02G448900		encodes Heparan-alpha-glucosaminide N-acetyltransferase	AT5G27730 (AT5G27730)
TRAESCS1B02G181800		encodes Alpha-galactosidase	AGAL2 (AT5G08370)
TRAESCS1B02G224600	FMOGS-OX1, FMOGS-OX3, FMOGS-OX4, FMOGS-OX5	encodes Flavin-containing monooxygenase FMO GS-OX-like 3	AT1G12130 (AT1G12130), FMO GS-OX5 (AT1G12140), AT1G12160 (AT1G12160)
TRAESCS2A02G048600		encodes Putative methyltransferase DDB_G0268948	AT2G41380 (AT2G41380)
TRAESCS1D02G121500		encodes LEA-like protein WSH1	
TRAESCS4B02G398400	M3KE1	encodes Serine/threonine-protein kinase fray2	AT4G24100 (AT4G24100)
TRAESCS1A02G064100	IQD1	encodes Protein IQ-DOMAIN 1	IQD1 (AT3G09710), iqd2 (AT5G03040)
TRAESCS4A02G142700		encodes Protein LURP-one-related 11	
TRAESCS3B02G131100	HSP17.7	encodes 17.8 kDa class II heat shock protein	HSP17.6I (AT5G12020), HSP17.6A (AT5G12030)
TRAESCS2B02G470400		encodes uncharacterized protein LOC123046642	
TRAESCS3B02G174500		encodes uncharacterized protein LOC123069291	

Sonalika transgenerational memory genes top correlated with the traits			
TRAESCS2A02G358900	PDP	encodes 2,3-dimethylmalate lyase	AT2G43180 (AT2G43180)
TRAESCS5B02G337300	M3KE1, STY17, STY8	encodes Serine/threonine-protein kinase STY17	STY8 (AT2G17700), STY17 (AT4G35780), STY46 (AT4G38470)
TRAESCS3B02G484000	BGLU5	encodes Beta-glucosidase 5	
TRAESCS4A02G486400		encodes uncharacterized protein LOC123088592	AT2G46915 (AT2G46915)
TRAESCS5A02G267000		encodes AT-hook motif nuclear-localized protein 10	AT2G33620 (AT2G33620)
TRAESCS4A02G060700		encodes GTP-binding protein At2g22870	EMB2001 (AT2G22870)
TRAESCS5B02G136500		encodes Serine--tRNA ligase	SRS (AT1G11870)
TRAESCS2A02G106500	CIPK29	encodes CBL-interacting protein kinase 29	CIPK4 (AT4G14580)
TRAESCS1B02G294300	HSC-2	encodes Heat shock 70 kDa protein 4	HSP70 (AT3G12580)
TRAESCS4A02G036200	4CLL1	encodes 4-coumarate--CoA ligase-like 1	AT4G05160 (AT4G05160)
TRAESCS1D02G030600	SARED1	encodes Uncharacterized protein At2g37660, chloroplastic	AT2G37660 (AT2G37660)
TRAESCS4A02G334400		encodes uncharacterized protein LOC123087609	AT1G28140 (AT1G28140)
TRAESCS1A02G200900		encodes Probable helicase MAGATAMA 3	AT1G65780 (AT1G65780), AT1G65810 (AT1G65810), AT4G05540 (AT4G05540)
TRAESCS4A02G168900	MHX1	encodes Magnesium/proton exchanger 1	MHX (AT2G47600)
TRAESCS2B02G554600		encodes Protein MIZU-KUSSEI 1	AT2G21990 (AT2G21990), AT4G39610 (AT4G39610)
TRAESCS1A02G110500		encodes hypothetical protein TRIUR3_24973	
TRAESCS5D02G397400		encodes Protein NAR1	NAR1 (AT4G16440)
TRAESCS3B02G017300		encodes Peroxisomal membrane protein PEX14	
TRAESCS4A02G159700	CGS1	encodes Cystathionine gamma-synthase 1, chloroplastic	MTO1 (AT3G01120)

TRAESCS1A02G339600		encodes Serine/threonine/tyrosine-protein kinase HT1	AT3G46930 (AT3G46930), AT5G58950 (AT5G58950)
